# Boosting Electrochemiluminescence of Carbon Nitrides via Molecular Capacitor‐Mediated Spatiotemporal Electron Coordination

**DOI:** 10.1002/advs.202506277

**Published:** 2025-10-29

**Authors:** Lingling Xiang, Yuhua Hou, Wang Li, Kaiqing Wu, Kaiyuan Wang, Yu Wang, Yanfeng Fang, Songqin Liu, Yanfei Shen, Yuanjian Zhang

**Affiliations:** ^1^ Jiangsu Engineering Laboratory of Smart Carbon‐Rich Materials and Device Jiangsu Province Hi‐Tech Key Laboratory for Bio‐Medical Research School of Chemistry and Chemical Engineering Southeast University Nanjing 211189 China; ^2^ Center of Clinical Laboratory Medicine Zhongda Hospital and Jiangsu Provincial Key Laboratory of Critical Care Medicine Medical School Southeast University Nanjing 210009 China; ^3^ Department of Oncology Zhongda Hospital Southeast University Nanjing 210009 China

**Keywords:** carbon nitride, electrochemiluminescence, electron accumulation, nitrogen deficiency, structure‐activity relationship

## Abstract

Carbon nitride (CN) enables non‐toxicity, low cost, high quantum efficiency, and tunable spectrum. Nevertheless, there co‐exists a timescale mismatch among kinetic steps of electrochemiluminescence (ECL) and a spatial competition of electrons between radiative recombination and interfacial redox reactions. Herein, a spatiotemporal coordination strategy is reported to enhance *Φ*_ECL_ of CN by molecular capacitor functionalization. Mechanism studies show the capacitor, consisting of N‐vacancies and −C≡N terminal groups, dynamically regulates electron capture and accumulation. Interestingly, the spatial confinement of accumulated electrons in molecular capacitors effectively enhances the radiative recombination probability. Meanwhile, the accumulated electrons construct a new pathway for fast electron transport, and the relaxation of the accumulated electrons coordinates the electron transfer in bulk CN and redox reactions at the electrode surface on the µs‐ms timescale, establishing temporal coordination across multiple time domains. As a result, the *Φ*_ECL_ of CN increases by up to 100 times, reaching 1480 times that of the standard Ru(bpy)_3_Cl_2_/K_2_S_2_O_8_ system. Accordingly, compared to pristine CN, the as‐developed ECL sensors using CN with molecular capacitor functionalization demonstrate significantly improved performance in the visual detection of nitrite ions (a typical environmental pollutant), for example, a 3600 fold lower detection limit and a 3‐order of magnitude broader detection linear range.

## Graphical abstract

Molecular capacitors are modified on carbon nitrides (CN) to dynamically regulate electron capture and accumulation in the defect states under different potentials. As a result, the electrochemiluminescence (ECL) efficiency increases by up to 100 times, owing to a new spatiotemporal coordination mechanism. It enables visual ECL testing of nitrite contaminants with significantly improved sensitivity and linear detection range.

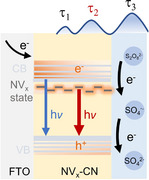

## 1 Introduction

The rapid development of modern industry and the growth of the global population call for highly efficient energy conversion to achieve sustainable social development. Among them, co‐reactant‐type electrochemiluminescence (ECL) is a process in which excited species generated by electrochemical reactions undergo energy transfer and emit light.^[^ ^1^, ^2^, ^3^, ^4^, ^5^ ^]^ For the exemplary system of Ru(bpy)_3_Cl_2_/K_2_S_2_O_8_, it mainly involves the reduction at the electrode surface, solution‐phase free radical‐mediated oxidation, annihilation between active species, and luminescence decay of the excited state.^[^ ^6^ ^]^ Owing to its high sensitivity, low background noise, and controllability, it has shown great potential and application prospects in diverse fields ranging from fundamental reaction kinetics studies to applied clinical diagnosis and environmental analysis.^[^ ^7^, ^8^, ^9^, ^10^, ^11^, ^12^, ^13^ ^]^ Generally, a high ECL efficiency (*Φ*_ECL_) plays a central role in the development of sensing systems with superior sensitivity. However, because ECL systems generally involve multiple charge transfer pathways, the complicated interactions at different time scales result in a low *Φ*_ECL_ for most ECL emitters in aqueous solutions, which significantly restricts the broader applications of ECL. For instance, the absolute anodic *Φ*_ECL_ of benchmark Ru(bpy)_3_Cl_2_ in acetonitrile under idealized conditions is only 5%, and that in aqueous solution is even lower by several orders of magnitude. After decades of development, few ECL emitters have surpassed this record; therefore, breaking the bottleneck of *Φ*_ECL_ is essential to pave the way for the next generation of ECL applications.^[^ ^6^, ^14^, ^15^ ^]^ Recently, covalently bonded carbon nitrides (CN) have emerged as a new generation of conjugated polymeric ECL emitters.^[^ ^16^ ^]^ The co‐reactant‐type ECL of CN involves four charge transfer pathways, namely electron injection, bulk electron transport, interfacial electron transfer, and electron‐hole radiative recombination, similar to that of Ru(bpy)_3_Cl_2_. By engineering of molecular orbit and interfacial electron transfer, the cathodic ECL efficiency of CN has increased to over 2000 times that of Ru(bpy)_3_Cl_2_/K_2_S_2_O_8_.^[^ ^13^, ^17^ ^]^

Charge carrier dynamics are central to the core and have a crucial impact on *Φ*_ECL_. Based on this principle, various innovative strategies have been proposed to improve one or more of these steps. For instance, decorating the interface with catalysts can improve the interfacial electron transfer efficiency,^[^ ^18^, ^19^, ^20^ ^]^ doping enhances charge transfer in the luminophore,^[^ ^21^, ^22^ ^]^ immobilizing or pre‐oxidizing the co‐reactant with emitters improves the hole injection efficiency,^[^ ^23^, ^24^, ^25^, ^26^ ^]^ and suppressing non‐radiative recombination boosts the generation of photons.^[^ ^27^ ^]^ However, it is worth noting that, similar to photosynthesis in nature, there is not only a huge time scale mismatch among the steps in the ECL process but also a spatial competition of electrons between radiative recombination and interfacial redox reactions. From a holistic perspective, developing a biomimetic spatiotemporal coordination strategy is of great significance for improving ECL efficiency, but it remains challenging.

Herein, we report a spatiotemporal coordination strategy to enhance *the Φ*_ECL_ by molecular capacitor functionalization. For this purpose, carbon nitrides were used as an example, and N vacancies and −C≡N terminal groups were introduced into the CN framework to form NV_x_‐CN. Comprehensive characterization techniques, including open‐circuit potential (OCP), distribution of relaxation times (DRT), electrochemical impedance spectroscopy (EIS), and time‐resolved photoluminescence, revealed the dynamic regulation of electron capture and accumulation by the molecular capacitors. Interestingly, the spatial confinement of the accumulated electrons in the molecular capacitor increased the probability and dynamics of radiative recombination. Meanwhile, the accumulated electrons construct a new pathway for fast electron transport, and the relaxation of the accumulated electron coordinates the electron transfer in bulk CN and redox reaction at the electrode surface in the µs‐ms timescale, ultimately achieving the optimization of multifaceted timescales. As a result, a significant improvement in *Φ*_ECL_ up to 100 times that of pristine CN was achieved.

## 2 Results and Discussion

### 2.1 Synthesis and Structural Characterization of CN and NV_x_‐CN

**Figure 1:**
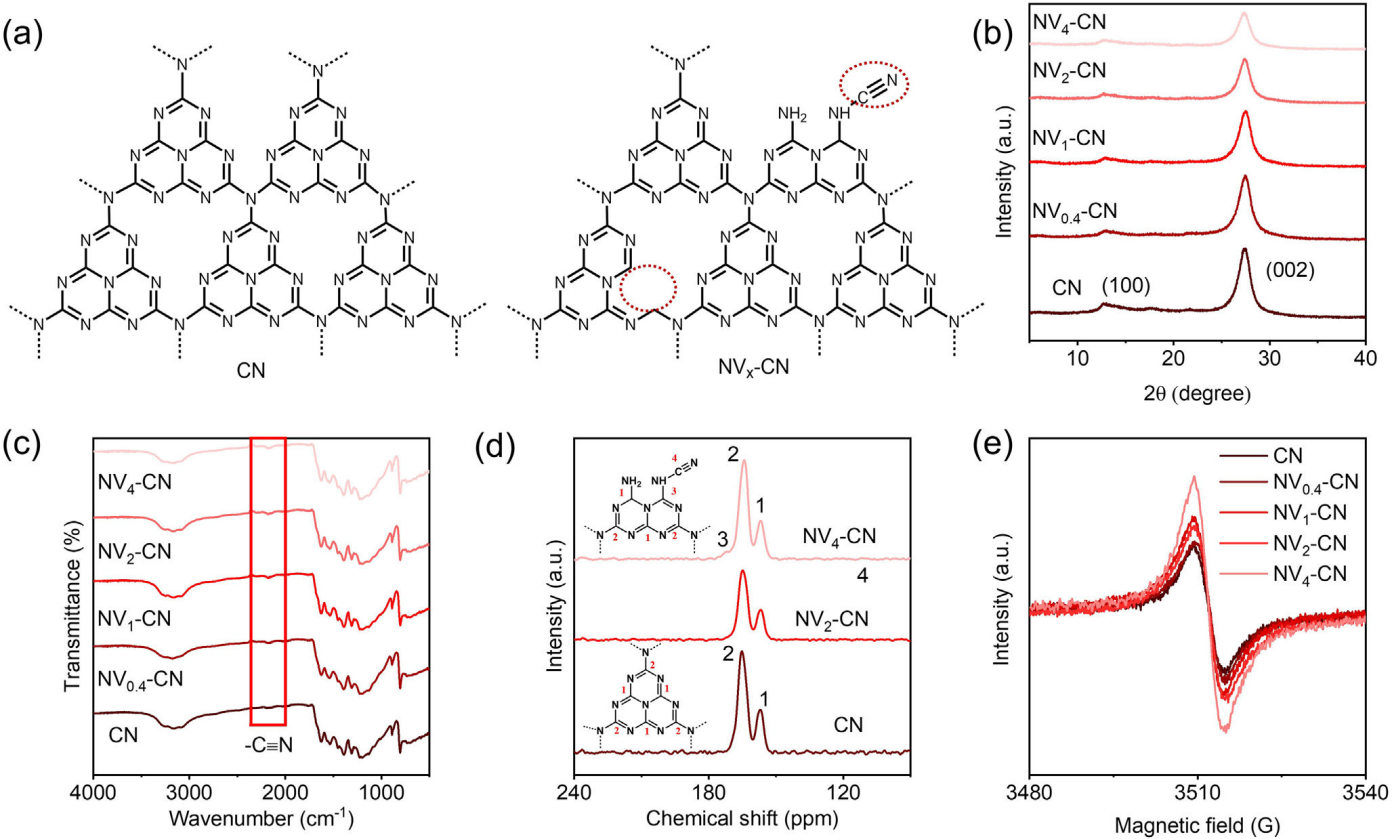
Structural characterization of CN and NV_x_‐CN. a) Structures of CN and NV_x_‐CN. XRD b) and FT‐IR c) spectra of CN, NV_0.4_‐CN, NV_1_‐CN, NV_2_‐CN and NV_4_‐CN. Solid‐state ^13^C MAS NMR d) and ESR e) spectra of CN, NV_0.4_‐CN, NV_1_‐CN, NV_2_‐CN, and NV_4_‐CN.

ECL systems based on emerging conjugated polymeric CN enable excellent photophysical stability, inherent biocompatibility, superior quantum efficiency, and remarkable structural modulation,^[^ ^28^, ^29^, ^30^, ^31^, ^32^ ^]^ which make them show great potential and application prospects in chemical measurements and clinical diagnosis. Here, NV_x_‐CN with N vacancies and −C≡N end groups was prepared as the model system to coordinate the carrier kinetics of each ECL step for CN. Pristine CN and NV_x_‐CN were prepared. Briefly, CN was synthesized using urea as the precursor via thermal condensation, while NV_x_‐CN was prepared by adding different proportions of KOH to the urea precursor (x represents the mass ratio of KOH used with 5 g of urea, ‰).^[^ ^33^ ^]^ It is evident that the KOH doping level in this work remains at a low value, which results in a low content of defects induced. Notably, the quantitative measurement of defect group density in NV_x_‐CN is anticipated. However, the accurate quantitative measurement of defects is still a significant challenge.^[^ ^33^, ^34^ ^]^ In this study, we revealed the structures of CN and NV_x_‐CN (**Figure** **1**a) based on the trend of structural evolution, and the detailed analysis of the related structural characterization is provided below.

The crystalline structure of the carbon nitride was investigated using X‐ray diffraction (XRD). The XRD patterns in Figure 1b display two typical diffraction peaks at 13.0° and 27.4°, which are attributed to the (100) in‐plane ordered arrangement of tri‐s‐triazine units and the (002) interlayer packing of CN aromatic units, respectively.^[^ ^32^, ^35^ ^]^ Both peaks gradually decreased with increasing KOH content, proving that the KOH content can regulate the defect content. This may result from the reaction of KOH with CN (or its intermediates) during thermal polymerization, which results in partial structural disorder. The detailed chemical structures of the different CN and NV_x_‐CN samples were studied using Fourier‐transform infrared spectroscopy (FT‐IR), as shown in Figure 1c. All the FT‐IR spectra of CN and NV_x_‐CN showed peaks near 800 and 1100–1700 cm^−1^, which were generally attributed to the in‐plane bending of the triazine or heptazine ring and C‐N heterocycle stretching vibration, respectively. It is worth noting that an asymmetrical stretching vibration of −C≡N at 2200 cm^−1^ was observed for NV_x_‐CN.^[^ ^35^ ^]^ Solid‐state ^13^C magic‐angle spinning (MAS) NMR measurements provided further insights into the structure of NV_x_‐CN. All the NMR spectra of the samples (Figure 1d) exhibited two strong peaks at 158.4 and 168.5 ppm, corresponding to the chemical shifts of C_3N_ (2) and C_2N–NHx_ (1) in the heptazine unit, respectively.^[^ ^36^ ^]^ Two new peaks were observed at 118.3 and 170.7 ppm, which can be assigned to the carbon atoms in the cyano group (4) and the C atom adjacent to the cyano group (3), respectively.^[^ ^37^ ^]^ Notably, the peak at 118.3 ppm was weak, but its existence can be verified by the vibration peaks for cyano groups in the FT‐IR spectrum (Figure 1c). This observation aligns with the low defect content noted earlier, making quantitative characterization of the 118.3 ppm peak challenging. Our primary goal for the ^13^C MAS NMR analysis was to identify cyanide sites; thus, the 170.7 ppm peak remains the key focus of our characterization. The above results jointly corroborate that the incorporation of KOH weakens the structural ordering degree of the tri‐s‐triazine/heptazine framework and successfully induces the construction of cyano groups (−C≡N).

To further study the effect of KOH treatment on the elemental composition of CN, X‐ray photoelectron spectroscopy (XPS) and organic elemental analysis (EA) measurements were performed, and the results are presented in Tables S1 and S2 (Supporting Information). Only trace amounts of oxygen were detected by EA, which excluded the possibility of oxygen‐containing functional groups being introduced by KOH treatment. The O1s peak in the XPS spectra may be due to surface‐adsorbed oxygen‐containing substances. They consistently indicated that the C/N ratio increased with increasing KOH content, suggesting more N defects. To further confirm the introduction of N defects, the narrow‐scan C1s and N1s XPS spectra of CN and NV_2_‐CN were deconvoluted into their components, as shown in Figure S1 and Table S3 (Supporting Information). The intensity of the N_2C_ peak of NV_2_‐CN was weaker than that of CN, indicating the formation of an N_2C_ vacancy in NV_x_‐CN.^[^ ^33^, ^38^ ^]^ These results provide strong evidence for the successful introduction of nitrogen defects (N_2C_) into the CN framework via a facile solid‐state thermal reaction of urea with KOH.

To indicate the tunable defect density in NV_x_‐CN, the defects were analyzed using electron paramagnetic resonance (ESR) spectroscopy to detect the signal changes generated by unpaired electrons and the surrounding structural features. The ESR spectra of CN, NV_0.4_‐CN, NV_1_‐CN, NV_2_‐CN, and NV_4_‐CN (Figure 1e) exhibit a single Lorentzian line.^[^ ^39^, ^40^ ^]^ The number of unpaired electrons increased in NV_x_‐CN with a high KOH content compared to that in CN, which proved the increase in defects.^[^ ^41^ ^]^ It also showed that the density of nitrogen defects can be regulated by the KOH content. The surface morphologies of CN and NV_2_‐CN were measured using scanning electron microscopy (SEM, Figure S2, Supporting Information). Considering that only a minimal number of defects were introduced, they exhibited negligible differences on their surfaces apart from variations in deepening color by the naked eye (Figure S3, Supporting Information).

### 2.2 ECL of CN and NV_x_‐CN Photoelectrodes

**Figure 2:**
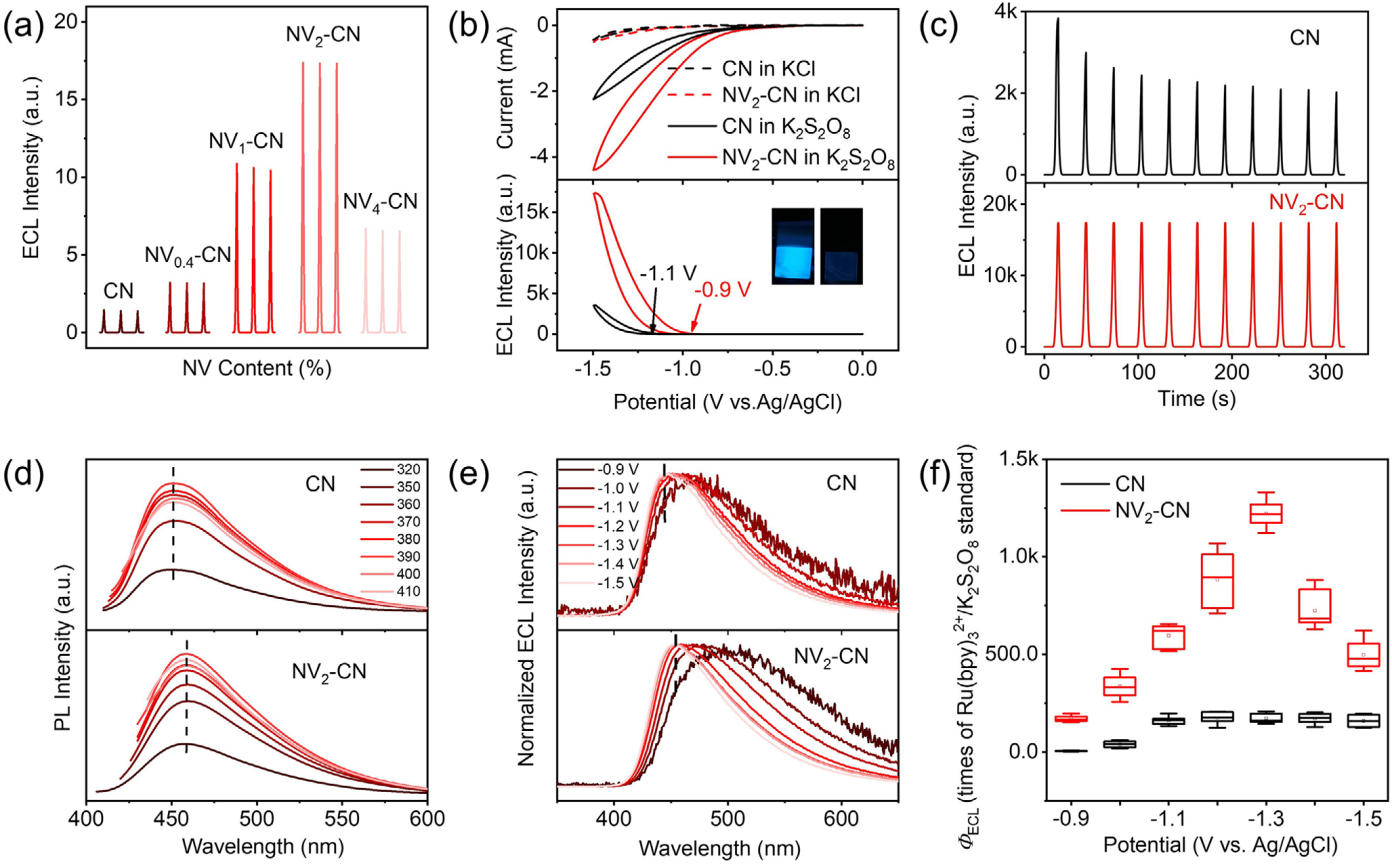
ECL performance of NV_x_‐CN photoelectrode. a) ECL intensity of NV_x_‐CN photoelectrodes with various NV contents in preparation under repetitive measurements. b) CV and ECL curves of the CN and NV_2_‐CN photoelectrodes. Electrolytes: 0.01 m phosphate‐buffered saline containing 0.1 M KCl with or without 15 mM K_2_S_2_O_8_. “a.u.” refers to arbitrary units. c) ECL stability of CN and NV_2_‐CN under continuous CV scan. d) PL spectra of CN and NV_2_‐CN under different excitation light wavelengths from 320 to 410 nm. e) Normalized ECL intensities of CN and NV_2_‐CN at different biased potentials. f) *Φ*_ECL_ values of CN and NV_2_‐CN at different potentials.

To explore the influence of defects on the ECL performance of CN, the ECL signals of CN and NV_x_‐CN with different defect concentrations were studied in a K_2_S_2_O_8_ solution. CN and NV_x_‐CN photoelectrodes were prepared by electrophoretic deposition (Figure S4, Supporting Information). To exclude the effect of the photoelectrode thickness, the same electrophoretic deposition time was roughly maintained to obtain the same photoelectrode thickness (Figure S2c,d, Supporting Information). As shown in **Figures** **2**a and S5 (Supporting Information), a typical volcano type was obtained for the ECL intensity of CN with the increase in defect content. Among them, the NV_2_‐CN photoelectrode exhibited the highest ECL intensity. The cyclic voltammetry (CV) curves of the CN and NV_2_‐CN photoelectrodes are shown in Figure 2b. Minor background currents (dashed lines) were observed for both the CN and NV_2_‐CN photoelectrodes in the solution without K_2_S_2_O_8_, indicating negligible polarization of water during the reduction of K_2_S_2_O_8_. Notably, the reduction peak of K_2_S_2_O_8_ is outside the electrochemical window owing to the high iR drop of the CN and NV_2_‐CN photoelectrodes. Compared with that of the CN photoelectrode, the ECL onset potential of the NV_2_‐CN photoelectrode shifted positively by 200 mV and exhibited a larger cathodic current, and the ECL intensity reached almost 8 times that of the CN photoelectrode. The strong emissions of NV_2_‐CN can be easily observed by the naked eye, and the uneven luminescence on the photoelectrodes may be due to their surface roughness. The ECL stability of CN and NV_2_‐CN was revealed in Figure 2c; CN decays to 60% of its original value after 200 s, while NV_2_‐CN has almost no attenuation. It was obvious that NV_2_‐CN exhibited higher stability of ECL signals after continuous CV scanning.

Figure 2d shows the PL spectra of CN and NV_2_‐CN under different excitation wavelengths ranging from 320 to 410 nm. All PL peak positions were independent of the excitation wavelength, indicating that the same excitation and recombination processes occurred in CN and NV_2_‐CN.^[^ ^42^ ^]^ As shown in Figure S6b (Supporting Information), the maximum PL wavelength of CN exhibited an ordered redshift (from 450 to 460 nm) with an increase in the defect concentration. This is in good agreement with the UV–vis spectra (Figure S7a, Supporting Information) and the band gap reduction (from 2.85 to 2.74 eV) shown by the band gap transformed using the Kubelka–Munk function (Figure S7b, Supporting Information), which further confirms that the PL of CN and NV_x_‐CN involves bandgap edge radiative recombination.

To gain insight into the ECL luminescence types of CN and NV_x_‐CN, the ECL spectrum was employed to collect continuous ECL spectra during a dynamic potential process. As shown in Figure 2e, the strong redshift of the ECL spectrum of NV_2_‐CN at low reduction potentials indicates that more states are produced below the edge of the NV_2_‐CN band.^[^ ^43^ ^]^ This phenomenon can be ascribed to radiative recombination associated with the introduction of defect‐trapping states. Similar peak wavelengths of ≈450 and 460 nm for CN and NV_2_‐CN, respectively, were observed near–1.50 V, which was in accordance with their PL spectra. Therefore, in contrast to the PL emission of NV_2_‐CN, which originates entirely from bandgap radiative recombination, the ECL of NV_2_‐CN also involves a small portion of defect‐state radiative recombination.

Notably, redshift of ECL under different biased potentials with respect to PL was observed (Figure 2e). By comparing the ECL spectra of CN and NV_2_‐CN, which were both subjected to bias potentials, we observed a more evident redshift in the ECL spectrum of NV_2_‐CN. Since CN and NV_2_‐CN had roughly similar geometric structure, it excluded the influence of the Stark effect–a redshift of the interband transmission under an external electric field. We speculated that it might more likely originate from the radiative recombination of edge defect states. On the one hand, differences in defect concentration or even type may lead to different defect energy levels, and the radiative recombination of different defect energy levels causes a broad spectral redshift. On the other hand, the difference between PL and ECL may be due to the different electron injection kinetics in the process of light excitation vs electrical injection. Different electron injection kinetics are sensitive to different energy levels, leading to different contributions to PL vs ECL. This is just as shown in the ECL spectra of NV_2_‐CN at different voltages in Figure 2e, i.e., the smaller the voltage, the smaller the electron injection rate, and the ECL spectrum shows a gradual redshift with the decrease of voltage. Nonetheless, more comprehensive future investigation is needed to fully understand the mechanism of redshift of ECL under different biased potentials.

Based on the above results and previous studies, the probable electron transfer processes of NV_x_‐CN were proposed.^[^ ^29^ ^]^ ECL is a light emission that results from an electron transfer reaction between electrochemically generated species near the electrode. In general, the entire ECL process of the NV_x_‐CN photoelectrode can be divided into four steps. First, electrons are injected from the substrate electrode into the conduction band (CB) of NV_x_‐CN to form NV_x_‐CN^•−^ (Equation 1). Then, some excited electrons in NV_x_‐CN^•−^ are captured by S_2_O_8_^2^^−^ and produce a strong oxidant SO_4_^•−^ (Equation 2), followed by an additional single‐electron extraction to produce a hole in the valence band (VB, Equation 3). Finally, the electrons in the CB and holes in the VB recombine to emit light (Equation 5).

(1) $$NV_{x}-\mathrm{CN}+e^{-}\to NV_{x}-CN^{•-}$$

(2) $$S_{2}{O_{8}}^{2-}+NV_{x}-CN^{•-}\to S{O_{4}}^{2-}+S{O_{4}}^{•-}+NV_{x}-\mathrm{CN}$$

(3) $$NV_{x}-CN^{•-}+S{O_{4}}^{•-}\to NV_{x}-CN^{*}+S{O_{4}}^{2-}$$

(4) $$\left(S{O_{4}}^{•-}\to S{O_{4}}^{2-}+h^{+}\right)$$

(5) $$NV_{x}-CN^{*}\to NV_{x}-\mathrm{CN}+\mathrm{hv}$$

The *Φ*_ECL_ values of CN and NV_2_‐CN at different potentials were evaluated using a readily reproducible Ru(bpy)_3_^2+^/K_2_S_2_O_8_ aqueous solution system as a standard reference. The details of the ECL efficiency measurement are provided in the experimental procedures of the Supporting Information. As shown in Figure 2f, the *Φ*_ECL_ of the CN photoelectrodes increased with increasing potential and reached a plateau at −1.1 V. Interestingly, *the Φ*_ECL_ of the NV_2_‐CN photoelectrodes first increased and then decreased with increasing potentials, reaching a maximum at −1.3 V. As a result, *the Φ*_ECL_ of the NV_2_‐CN photoelectrodes was 4–100 times higher than that of CN (see the detailed calculation of the corrected number of photons and electrons in Figures S8–S10, Supporting Information), ≈1480 times that of the aqueous Ru(bpy)_3_^2+^/K_2_S_2_O_8_ reference system at −1.3 V. To understand the mechanism of the maximum *Φ*_ECL_ at −1.3 V, the charge carrier dynamics at different potentials during the multiple processes of ECL were explored and discussed in the following context.

### 2.3 Electron Accumulation by Molecular Capacitors in NV_x_‐CN

**Figure 3:**
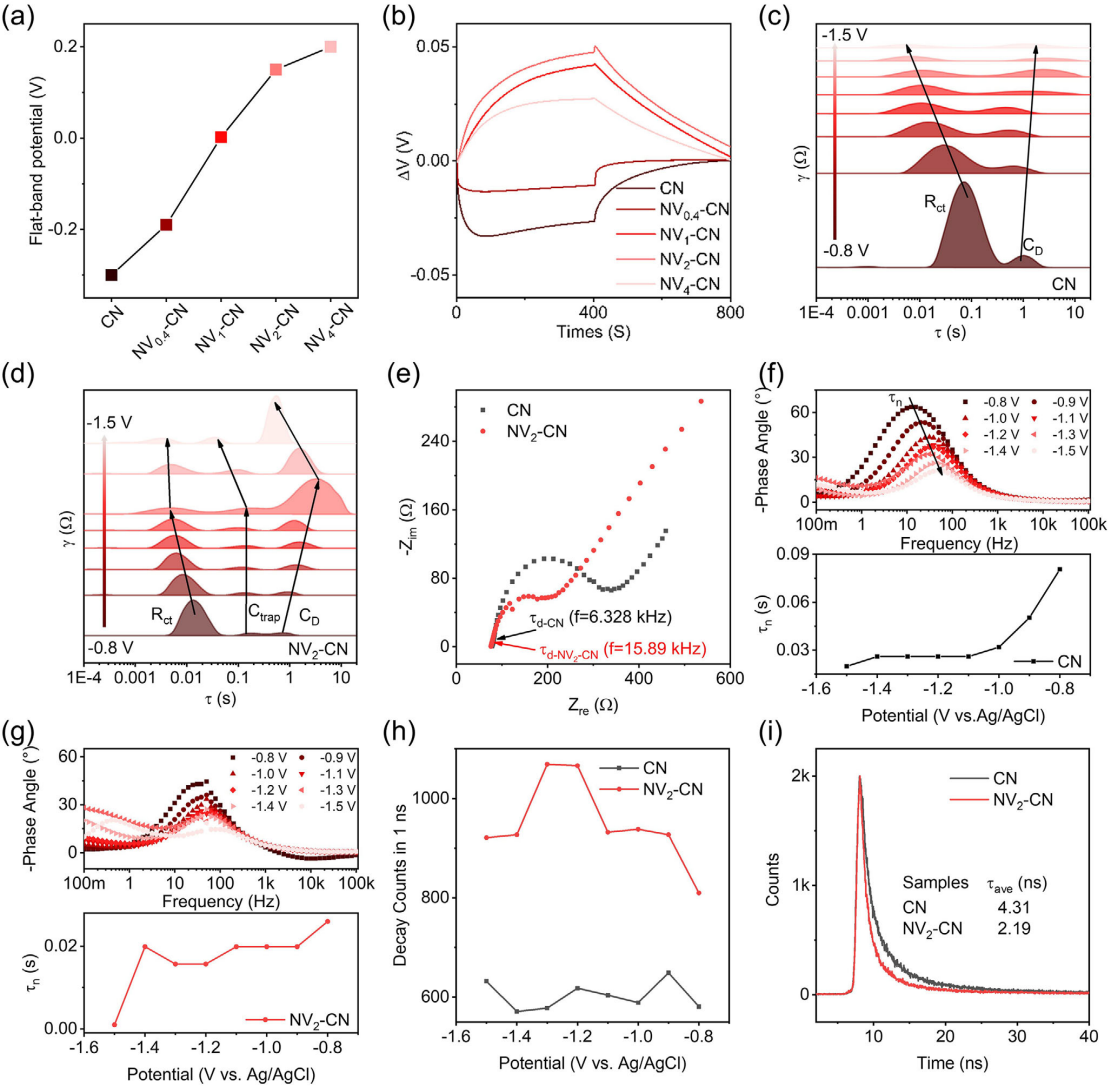
Electron transfer kinetics of NV_x_‐CN. a) Flat‐band potential of the CN and NV_x_‐CN photoelectrodes. b) Open‐circuit potential of CN and NV_x_‐CN photoelectrodes under chopped visible light in 0.1 M KCl. DRT fitting data of EIS for the CN c) and NV_2_‐CN d) photoelectrodes at different potentials. e) Nyquist plots for the CN and NV_2_‐CN photoelectrodes at −1.3 V. Electrolytes: 0.01 m phosphate‐buffered saline containing 0.1 M KCl and 15 mM K_2_S_2_O_8_. Bode phase plots and characteristic lifetime τ_n_ extracted from the medium frequency EIS spectra of the CN f) and NV_2_‐CN g) photoelectrodes at different potentials in 0.01 M phosphate‐buffered saline containing 0.1 m KCl and 15 mM K_2_S_2_O_8_. h) Integrated counts of electron decay at different potentials under 1 ns of light excitation. i) Time‐resolved PL decay spectra of CN and NV_2_‐CN under 360 nm excitation with a biased potential of −1.3 V.

To verify the mechanism of the enhanced ECL, LSV curves (Figure 3a; Figure S11, Supporting Information) of CN and NV_x_‐CN photoelectrodes under chopped light were first measured. Compared with CN, the flat band potential of NV_x_‐CN continuously shifted positively as the defect concentration increased. This observation can be ascribed to the defect states that captured and accumulated electrons, changed the built‐in electric field, and mediated band bending.^[^ ^44^ ^]^ Moreover, the ability of CN to accumulate electrons increased with increasing defect concentration. The OCP plot (Figure 3b) demonstrates similar results. A negative photovoltage, indicative of the typical behavior of n‐type semiconductors, was observed under illumination. As the defect concentration increased, the photovoltage became positive, suggesting that the increase in accumulated electrons caused the semiconductor type to switch from n‐type to p‐type.^[^ ^45^ ^]^ Notably, for NV_4_‐CN with the maximum concentration of defects, the p‐type characteristic weakened, which was ascribed to the asymmetric dynamics between electron capture and release (Figure S12 and Table S4, Supporting Information).^[^ ^46^ ^]^ Therefore, the defects, including N‐vacancies and −C≡N terminal groups in carbon nitride, captured and accumulated electrons, which can be regarded as molecular capacitors.

To understand the effect of electron accumulation at different potentials during the ECL process, EIS, which is commonly used to study charge carrier kinetics, such as charge transport and recombination, was further explored. However, in practical data analysis, the carrier kinetics processes of complex systems may overlap with the impedance spectrum, making them difficult to distinguish,^[^ ^47^, ^48^ ^]^ as shown in Figure S13 (Supporting Information). To understand the influence of accumulated electrons on charge carrier dynamics, it is important to understand its variation trend. For this purpose, DRT^[^ ^49^, ^50^, ^51^, ^52^ ^]^ was used to fit the EIS data of CN and NV_x_‐CN at various potentials. The EIS data in the frequency domain were converted into impedance distribution data in the time domain so that processes with different kinetic time constants but overlapping in the impedance spectrum were decoupled in the time scale.^[^ ^53^, ^54^ ^]^ The EIS‐DRT graphs at different potentials are shown in **Figure** **3**c,d. Different characteristic peaks represent different relaxation processes for the charge carriers in the system. The electronic dynamics can be directly monitored by assigning characteristic peaks to specific processes and following their evolution during ECL. The key to analyzing the attribution of the characteristic peaks is to analyze the capacitance parallel to the resistance in the system.

Generally, the original CN contains two capacitances: the double‐layer capacitance (C_d_), which controls the interfacial charge transfer resistance (R_ct_) during the Faraday reaction on the millisecond timescale, and the diffusion layer capacitance (C_D_), which controls the Warburg impedance on the second timescale. However, NV_x_‐CN exhibited a third type of capacitance due to the accumulated electrons in the defects, which is referred to as the trap capacitance (C_trap_). Its expression is given by Equation (6):^[^ ^55^, ^56^ ^]^

(6) $$C_{trap}=A_{S}qN_{ss}\frac{\partial f_{ss}}{\partial E_{Fn}}=A_{S}\frac{N_{ss}q^{2}}{k_{B}T}f_{ss}\left(1-f_{ss}\right)$$

where N_ss_ is the surface density of the surface states, f_ss_ is the fractional occupancy of the state, and E_Fn_ is the electronic Fermi level of the state. It can be inferred from the formula that as the electron injection level increases, C_trap_ exhibits a peak at E_Fn_ = E_ss_, where E_ss_ is the energy level of the surface state. This trend is consistent with the unique feature peak changes in the millisecond‐to‐second relaxation time scale in NV_x_‐CN (Figure 3d), which first increased and then decreased with increasing potentials and reached a maximum at −1.3 V. (see fitting parameters in Table S5, Supporting Information). The approximate equivalent circuit used for the interpretation of the CN and NV_x_‐CN photoelectrodes in the DRT fitting is shown in Figure S14 (Supporting Information).

The quantitative measurement of accumulated electrons by the traditional chemical titration method is promising. However, typical electron acceptors, such as Ag^+^ or methylviologen^2+^, would directly obtain electrons from the substrate electrode under different reduction potentials in this study, leading to a large background, while the number of the accumulated electrons from charged CN is much smaller. To address this limitation, the electron accumulation characteristics of the same material under different potentials were characterized through DRT fitting analysis in this study (Figure 3d; Table S5, Supporting Information).

### 2.4 Effects of Electron Accumulation on Bulk Electron Transport

To corroborate the impact of the accumulated electrons on bulk electron transport, the carrier diffusion lifetime τ_d_ and electron mobility μ of the CN and NV_x_‐CN photoelectrodes were quantitatively measured by EIS (Figure 3e). The inflection point of the straight line of the high‐frequency part and the arc is related to τ_d_, which is inversely correlated with the frequency.^[^ ^57^ ^]^ The calculated τ_d_ values of the CN and NV_x_‐CN photoelectrodes were 159 and 62.9 µs, respectively. The electron mobility was calculated using the Nernst–Einstein equation (Equation 7):^[^ ^58^, ^59^ ^]^

(7) $$\mu =\frac{eL^{2}}{k_{B}T\tau_{d}}$$

The electron mobility of the NV_2_‐CN photoelectrode was calculated to be 5.57 × 10^−2^ cm^2^ V^−1^ s^−1^, which is 2 times that of the CN photoelectrode (2.20 × 10^−2^ cm^2^ V^−1^ s^−1^). The decreased τ_d_ value and increased electron mobility indicate that the diffusion kinetics of electrons in the NV_x_‐CN photoelectrode are faster. This is consistent with the results from EIS measurements using the fast redox couple Fe(CN)_6_^3−^/Fe(CN)_6_^4−^ as the electrochemical probe (Figure S15, Supporting Information), which demonstrated that the defect‐accumulated electrons broke the energy barrier of the defective capacitor (E_trap_) and promoted charge transport in the bulk emitter (see more discussion on higher electron mobility in defect CN in the Supporting Information)^[^ ^60^, ^61^ ^]^

### 2.5 Effects of Electron Accumulation on Interfacial Electron Reaction

The impact of electron accumulation on interfacial reactions was confirmed via DRT analysis of the EIS data. As shown in Figure 3c,d, for the pristine CN, R_ct_, and the corresponding relaxation time constants exhibited a continuous decline with increasing applied potentials, directly evidencing enhanced interfacial reaction kinetics governed by potential‐driven charge transfer dynamics. Notably, for NV_2_‐CN, C_trap_ and the corresponding relaxation time constants first increased and then decreased with increasing potentials, reaching a maximum at −1.3 V. This coincides with the turning point of R_ct_ and C_D_, suggesting that the longer the relaxation times of the accumulated electrons, the more coordinated the interfacial electron reaction time scale, which ultimately leads to a smaller R_ct_ and larger C_D_ in the solution.^[^ ^62^, ^63^ ^]^

In addition, the phase angle vs frequency plots at different potentials of the CN and NV_2_‐CN photoelectrodes were used to probe the quantitative reduction kinetics of the emitter/S_2_O_8_^2−^ during the ECL process. The peaks in the medium‐frequency region are related to the effective lifetime of the electrons (τ_n_). It was observed that the τ_n_ of the CN photoelectrode gradually decreased with increasing reduction potentials (Figure 3f), indicating that the external potential can promote the interfacial reaction. Interestingly, as shown in Figure 3g, for the NV_2_‐CN photoelectrode at the same potential, τ_n_ was only half of that of the CN photoelectrode, and a peak value of τ_n_ appeared at −1.3 V, where the electron accumulation reached the maximum value (Figure 3d). These results show that, except for the external potential, τ_n_ can also be regulated by the accumulated electrons. Moreover, a shorter τ_n_ is associated with faster interfacial electron transfer kinetics. Considering that more accumulated electrons have a longer relaxation time (Figure 3d), an increase in the number of accumulated electrons can coordinate the interfacial electron reaction timescale and promote interfacial electron transfer. In addition, from the Frequency‐Z_mod_ at different potentials for CN and NV_2_‐CN (Figure S16, Supporting Information), NV_2_‐CN has smaller resistances at the onset current potential, which may result from the lowered energy barrier of the interfacial reaction by the accumulated electrons. Therefore, the accumulation of electrons synergistically promotes interfacial charge transfer by coordinating the timescale of charge transfer in bulk CN and redox reactions at the electrode surfaces and lowering the interfacial charge transfer energy barrier.

### 2.6 Effects of Electron Accumulation on Radiative Recombination

Time‐resolved PL spectroscopy was used to study the effect of accumulated electrons on radiative recombination. In general, during ECL, electrons are continuously injected into the conduction band of CN or NV_2_‐CN at different potentials. To simulate the state of electron accumulation at defects, 360 nm pulsed excitation light and a constant different potential were applied, and the PL signal produced by CN or NV_2_‐CN was detected. Because the time scale of electron recombination is in the range of picoseconds to nanoseconds, the photon count of PL in 1 ns was integrated to compare the relative PL intensity.^[^ ^46^ ^]^ The integrated photon decay counts under different potentials are shown in Figure 3h. Notably, the changes in photon attenuation first increased and then decreased with the applied potentials, reaching a maximum at −1.3 V. This trend is the same as the relationship between the accumulated electrons and the biased potential (Figure 3d), suggesting that more accumulated electrons result in more attenuated photons. Furthermore, by fitting the decay curves, the PL lifetimes of CN and NV_2_‐CN (τ_ave_, Figure 3i) at −1.3 V were measured to be 4.31 and 2.19 ns, respectively. The complete fluorescence lifetime analysis of CN and NV_2_‐CN at other potentials is shown in Table S6 (Supporting Information). Compared to CN, NV_2_‐CN demonstrated an increased photon count and decreased PL lifetime. This indicates that more accumulated electrons were spatially confined in the molecular capacitance, which would increase the probability of radiation recombination.

### 2.7 Mechanism of Electron Accumulation by Molecular Capacitor for Boosting ECL Efficiency

**Figure 4:**
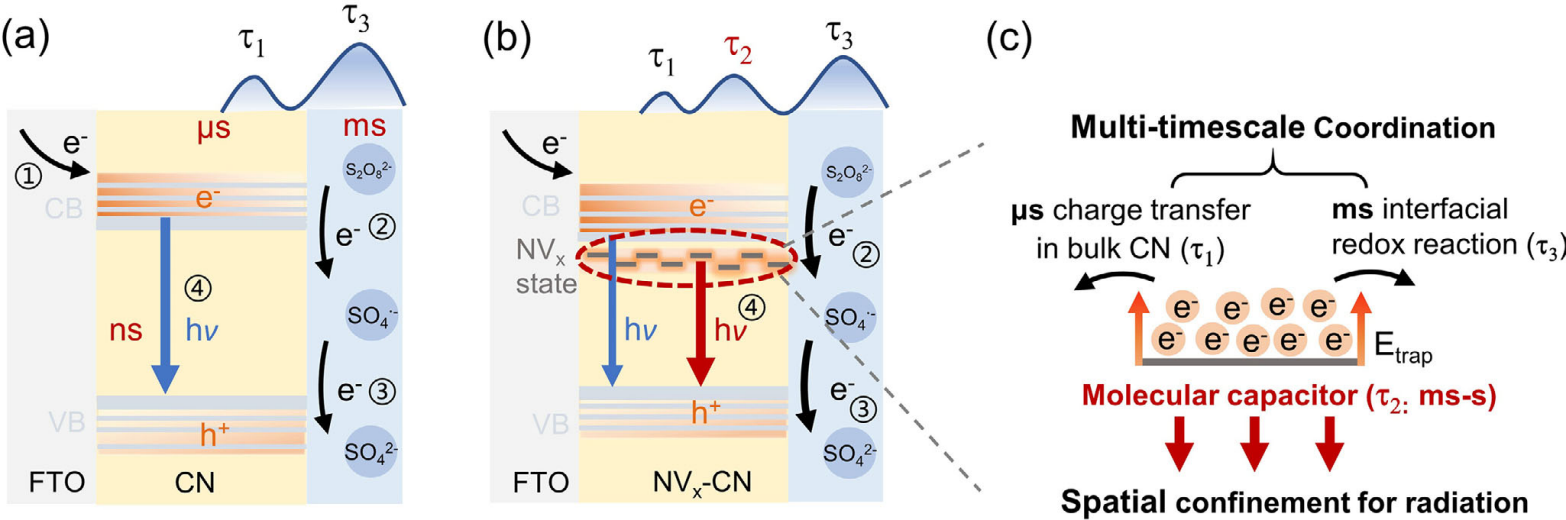
Charge transfer processes of ECL in CN a) and NV_x_‐CN b). Magnified view of the role of the NV_x_ state for charge transfer c). The ECL processes of “1‐4″ represent electron injection, bulk electron transport, interface electron transfer, and radiative recombination, respectively.

The quantitative values of the electron transfer kinetic parameters in the ECL processes of CN and NV_2_‐CN are summarized in **Table** **1**. The role of the molecular capacitor in the electron transfer dynamics during the ECL process of NV_x_‐CN is shown in **Figure** **4**. Briefly, upon electron injection, free electrons populate the conduction band of NV_x_‐CN, with a fraction captured by the molecular capacitor, forming defect states. In general, energy can be considered to be stored reversibly in a capacitor in the form of an entropy change. When a potential is applied, more electrons accumulate, and the metastable electrons would overcome E_trap_, undergoing entropy‐driven transfer to restore equilibrium,^[^ ^64^ ^]^ which is favorable for an accelerated electron transfer in bulk carbon nitride. Moreover, electron accumulation in the millisecond to second timescale in the molecular capacitor can coordinate the timescale of bulk electron transfer in microseconds and redox reactions in milliseconds at the electrode surface, ultimately achieving the optimization of multifaceted timescales. Meanwhile, the electron accumulation in the capacitor would confine electrons in space, which increases the probability and dynamics of radiative recombination. Therefore, the multi‐level and cross‐scale charge carrier dynamics in the ECL system were successfully regulated by a spatiotemporal coordination strategy using metastable electrons captured and accumulated by molecular capacitors.

**Table 1:** Kinetic parameters of electron transfer in the ECL of CN and NV_2_‐CN.

Electron transfer stage	Parameters	CN	NV_2_‐CN
Bulk ECL emitter	τ_d_ (µs)^a)^	159	62.9
	µ (cm^2^ V^−1^ s^−1^)^b)^	2.20 × 10^−2^	5.57 × 10^−2^
Emitter/co‐reactant interface	τ_n_ (ms)^c)^	19.9	9.9
Light emission	τ_l_ (ns)^d)^	4.31	2.19

^a)^ carrier diffusion lifetime,

^b)^ electron mobility,

^c)^ effective lifetime,

^d)^ electron‐hole recombination lifetime.

### 2.8 NV_x_‐CN‐Based ECL Sensors for Nitrite Detection with Enhanced Performance

**Figure 5:**
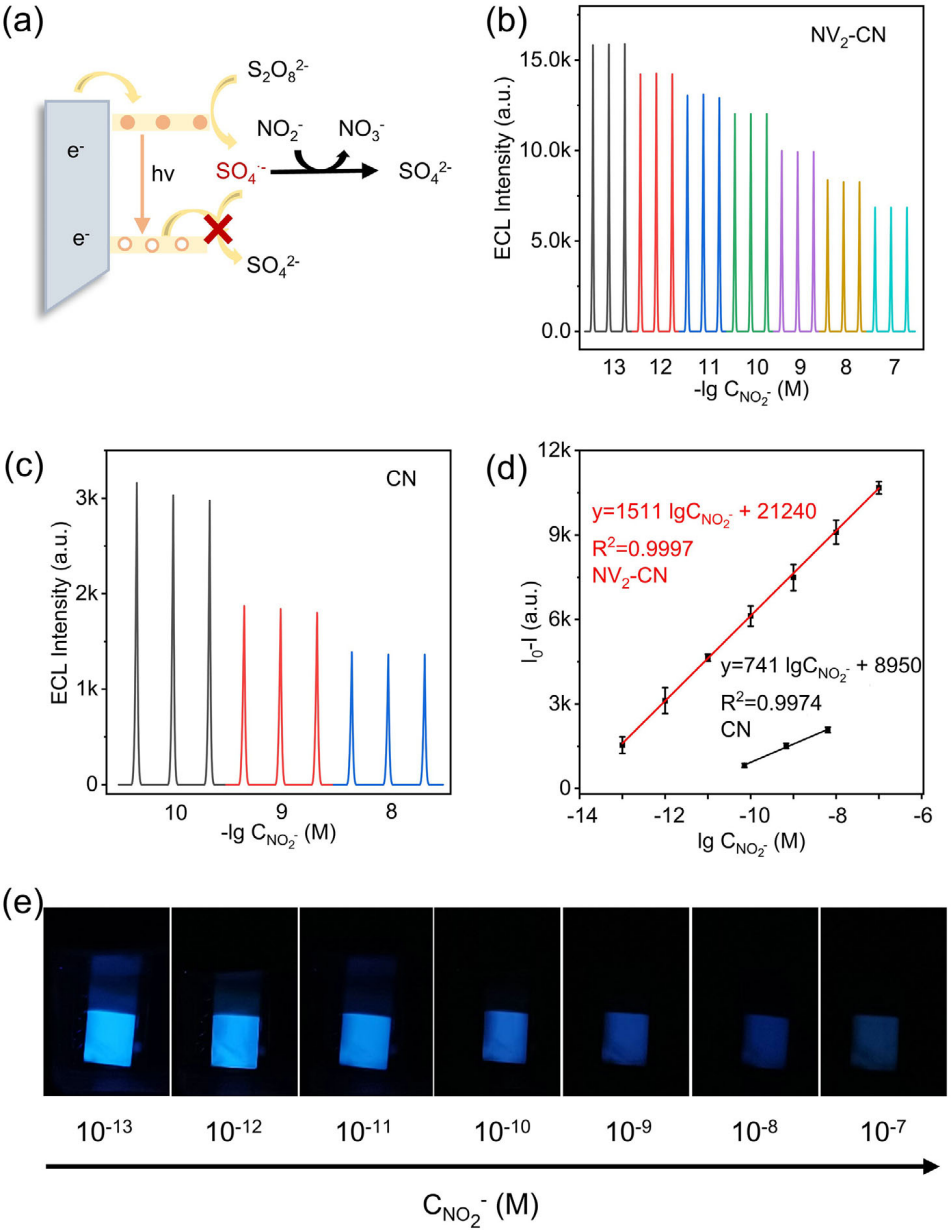
Nitrite sensors using CN and NV_2_‐CN. NO_2_^−^ detection mechanism based on ECL quenching a). ECL curves for different concentrations of NO_2_^−^ at NV_2_‐CN b) and CN c). Different colors indicate the concentrations indicated on the *x*‐axis. “a.u.” refers to arbitrary units. d) Calibration curve of NO_2_^−^ detection using NV_2_‐CN and CN. I_0_ and I are the ECL intensities before and after the addition of NO_2_^−^, respectively. Error bars represent the standard errors derived from three independent measurements. e) Photographs of ECL at NV_2_‐CN photoelectrode in solution containing different concentrations of NO_2_^−^ (see the ECL image without NO_2_^−^ in Figure 2b).

Nitrite, a key ingredient in fertilizers and food additives, is ubiquitous in the environment. Excessive concentrations of nitrite ions (NO_2_^−^) can have serious health effects on humans.^[^ ^65^, ^66^, ^67^ ^]^ Therefore, it is particularly important to establish a simple, flexible, and accurate method for the visual detection of nitrite, such as by the naked eye. Considering the high *Φ*_ECL_, an ECL visual biosensor based on NV_2_‐CN was developed for NO_2_^−^ determination (**Figure** **5**a). In principle, NO_2_^−^ can consume SO_4_^•−^ around the electrode surface, leading to a decreased ECL intensity.^[^ ^17^ ^]^ The cathodic ECL signals of the CN and NV_2_‐CN electrodes gradually decreased with an increase in NO_2_^−^ (Figure 5b,c). The logarithmic value of the ECL intensity of NV_2_‐CN had a linear relationship with the NO_2_^−^ concentration, which showed a wider detection range from 1 × 10^−13^ to 1 × 10^−7^ m and a lower detection limit (24.2 fM) compared to CN (1 × 10^−10^ to 1 × 10^−8^ m, 89.1 pM) (Figure 5d). Consequently, the detection limit of NV_2_‐CN is 3600 times lower than that of CN, and its linear detection range is 3‐orders of magnitude wider. To evaluate the reliability of this method, the stability of the proposed ECL sensing platform was assessed using continuous cyclic potential scanning. It demonstrated remarkable electrochemical stability, exhibiting negligible variation in performance over 10 consecutive cyclic potential scans (Figure S17, Supporting Information). Notably, owing to the extremely high cathodic ECL efficiency, visual cathodic ECL biosensors can be developed (Figure 5e).

It should be noted that the study of the NO_2_^−^ sensor serves only as an example of potential applications. For practical applications, concerns regarding selectivity should be well addressed. For example, future research will explore the attainment of multiple‐cycle detection of targets via dynamic regulation of potential and ECL signal. Additionally, machine learning will be employed to construct a “multi‐dimensional response signal‐interference correction” model to eliminate the potential interference on the target signal.

## 3 Conclusion

In summary, we present a molecular capacitor‐mediated spatiotemporal coordination strategy for improving ECL efficiency. Compared with the original CN, the ECL efficiency of the NV_x_‐CN photoelectrode increased by up to 100 times, reaching 1480 times that of the standard Ru(bpy)_3_Cl_2_/K_2_S_2_O_8_ system. Comprehensive characterization techniques such as LSV, OCP, DRT, EIS, and time‐resolved PL revealed the dependence of enhanced charge carrier dynamics on the accumulated electrons under different potentials. In general, N vacancies and −C≡N terminal groups can accumulate electrons to form a molecular capacitor, which adjusts the spatial distribution of electrons in bulk CN to increase the probability of radiation recombination. Meanwhile, accumulated electrons also establish a new pathway for fast electron transport and synchronized microsecond charge transfer in bulk CN with millisecond redox reactions at the electrodes, realizing the coordination of multifaceted time scales. As a proof‐of‐concept application, the NV_x_‐CN platform enabled ultrasensitive visual ECL detection of nitrite contaminants with 3600 fold lower detection limits and 3‐order of magnitude broader linear range than conventional CN. The excellent cathodic *Φ*_ECL_ endows this system with great potential as a platform to advance visually interpretable biosensing architectures.

## Conflict of Interest

The authors declare no conflict of interest.

## Supporting information



Supporting Information

## Data Availability

The data that support the findings of this study are available from the corresponding author upon reasonable request.
