# Supplementary material for: Boosting Electrochemiluminescence of Carbon Nitrides via Molecular Capacitor‐Mediated Spatiotemporal Electron Coordination
Source: Adv Sci (Weinh). 2025 Oct 29;13(3):e06277. doi: 10.1002/advs.202506277 (PMC12806443; doi:10.1002/advs.202506277)
Supplement: Supplementary file 1 — Supporting Information [file ADVS-13-e06277-s001.pdf]

Supplementary Information

Boosting Electrochemiluminescence of Carbon Nitrides via Molecular Capacitor-Mediated Spatiotemporal Electron Coordination

Lingling Xiang,[a] Yuhua Hou,[a] Wang Li,[a] Kaiqing Wu,[a] Kaiyuan Wang,[a] Yu Wang,[a] Yanfeng Fang,[a] Songqin Liu,[a] Yanfei Shen,[b] Yuanjian Zhang*[a,c]

[a]Jiangsu Engineering Laboratory of Smart Carbon-Rich Materials and Device, Jiangsu Province Hi-Tech Key Laboratory for Bio-Medical Research, School of Chemistry and Chemical Engineering, Southeast University, Nanjing 211189, China, E-mail: Yuanjian.Zhang@seu.edu.cn

[b]Center of Clinical Laboratory Medicine, Zhongda Hospital, and Jiangsu Provincial Key Laboratory of Critical Care Medicine, Medical School, Southeast University, Nanjing 210009, China, E-mail: Yanfei.Shen@seu.edu.cn

[c]Department of Oncology, Zhongda Hospital, Southeast University, Nanjing, 210009, China

Table of Contents

Name	Page
**Experimental section**	**4**
**Fig. S1** High-resolution N1s and C1s XPS spectra of CN, NV_2-CN and NV_4-CN.	**8**
**Fig. S2** Top and cross-sectional SEM images of CN (a and c) and NV_2-CN (b and d).	**9**
**Fig. S3** Digital photograph of samples prepared with different amounts of KOH.	**10**
**Fig. S4** Scheme of the general fabrication procedure for CN and NV_x-CN on the FTO glass.	**11**
**Fig. S5** ECL emission intensity of NV_x-CN photoelectrode with various KOH content.	**12**
**Fig. S6** PL spectra (a) and normalized PL spectra (b) of the CN, $NV_{0.4}$-CN, NV_1-CN, NV_2-CN and NV_4-CN.	**13**
**Fig. S7** UV-vis spectrum (a) and Kubelka-Munk plot (b) of the CN, $NV_{0.4}$-CN, NV_1-CN, NV_2-CN and NV_4-CN. VB-XPS of CN (c), NV_2-CN (d). (e) Energy level diagram of CN and NV_2-CN.	**14**
**Fig. S8** ECL intensity of CN (a) and NV_2-CN (b) photoelectrodes at different potentials.	**15**
**Fig. S9** Amperometric Q-t curve for ECL reaction of CN at –0.9 V (a), –1.0 V (b), –1.1 V (c), –1.2 V (d), –1.3 V (e), –1.4 V (f) and –1.5 V (g) in 0.01 M PBS, 0.1 M KCl with 15 mM $K_2S_2O_8$ (red line) and without $K_2S_2O_8$ (black line). (h) The number of charges consumed by Faraday reactions at different potentials (–0.9 V~–1.5 V).	**16**
**Fig. S10** Amperometric Q-t curve for ECL reaction of NV_2-CN at –0.9 V (a), –1.0 V (b), –1.1 V (c), –1.2 V (d), –1.3 V (e), –1.4 V (f) and –1.5 V (g) in 0.01 M PBS, 0.1 M KCl with 15 mM $K_2S_2O_8$ (red line) and without $K_2S_2O_8$ (black line). (h) The number of charges consumed by Faraday reactions at different potentials (–0.9 V~–1.5 V).	**17**
**Fig. S11** Current–potential curves for the CN (a), $NV_{0.4}$-CN (b), NV_1-CN (c), NV_2-CN (d), and NV_4-CN (e) photoelectrodes under chopped irradiation.	**18**

Fig. S12 Time-resolved PL decay spectra of CN, $NV_{0.4}$-CN, NV_1-CN, NV_2-CN and NV_4-CN under 360 nm excitation. **19**

Fig. S13 Impedance diagrams (Frequency-C_{real}) measured at different potentials for CN (a) and NV_2-CN (b). **20**

Fig. S14 Approximate equivalent circuit used for interpretation of CN and NV_x-CN photoelectrodes in DRT fitting. **21**

Fig. S15 Nyquist plots for CN and NV_2-CN in 0.1 M KCl containing 5 mM $K_3[Fe(CN)_6]/K_4[Fe(CN)_6]$. **22**

Fig. S16 Impedance diagrams (Frequency-Z_{mod}) measured at different potentials for CN (a) and NV_2-CN (b). **23**

Table S17 ECL intensity of NV_2-CN based biosensor used to detect NO_2^- under consecutive cyclic potential scanning. **24**

Table S1 Elemental analysis using the XPS data. **25**

Table S2 Elemental analysis (EA) results of CN, NV_2-CN and NV_4-CN. **26**

Table S3 The proportion of N_{2C}, N_{3C}, N-H_x, and charging effect in CN and NV_2-CN according to XPS N1s analysis. **27**

Table S4 Fitted τ_1, τ_2, and τ_3 using the Time-resolved PL decay spectra of CN, $NV_{0.4}$-CN, NV_1-CN, NV_2-CN and NV_4-CN under 360 nm excitation. **28**

Table S5 Summary of the impedance fitting data for CN and NV_2-CN photoelectrode. **29**

Table S6 Fitted τ_1, τ_2, τ_3, and τ_{ave} using the Time-resolved PL decay spectra of CN and NV_2-CN under 360 nm excitation at different potential. **30**

Discussion about higher electron mobility in defect CN **32**

Supplementary references **33**

Methods

Reagents

Urea (99%) was purchased from Sigma Aldrich. Potassium hydroxide (KOH), potassium peroxodisulfate ($K_2S_2O_8$), potassium chloride (KCl), sodium dihydrogen phosphate dihydrate ($NaH_2PO_4·2H_2O$), disodium hydrogen phosphate dodecahydrate ($Na_2HPO_4·12H_2O$), sodium nitrite ($NaNO_2$) and hydroiodic acid (HI) were obtained from Shanghai Macklin Biochemical Co. Ltd., China. Fluorine-dopedtinoxide (FTO) glass (12–14 Ω/sq, ZhuhaiKaivo Optoelectronic Technology Co., Ltd., China) was ultrasonically washed with acetone, ethanol, and ultrapure water for 15 min, respectively, and then dried with high purity nitrogen gas flow before use. Ultrapure water (18.2 MΩ·cm) was obtained from a Direct-Q 3 UV pure water purification system (Millipore, USA) throughout all experiments. Unless otherwise specified, all the other reagents were of analytical grade and used without further purification.

Characterization

UV-vis absorption spectra were measured on a Cary 100 (Agilent, Singapore) with a diffuse-reflectance accessory, and $BaSO_4$ was used as a standard reference (100% reflectance). Fourier-transformed infrared spectra (FTIR) were recorded using a Nicolet iS10 FTIR spectrometer, equipped with an attenuated total reflection (ATR) setup (Thermo, USA). Photoluminescence (PL) spectra and the time-resolved photoluminescence spectra (TRPL) were performed on Fluoromax-4 (Horiba Jobin Yvon, Japan). Scanning electron microscopy (SEM) images of CN and NV_x-CN was investigated by Sigma 360 (Zeiss, Germany). The XRD patterns were measured by using Ultima IV (Rigaku, Japan). X-ray photoelectron spectra (XPS) was taken on a Scientific K-Alpha electron spectrometer (Thermo, USA) with monochromatic Al Kα X-rays (hν = 1486.6 eV) as the excitation source, and the binding energy was corrected by reference C1 s level to 284.6 eV to compensate for the specimen charging. The C, N, and O contents were measured via an Organic element analyzer (EA). Solid-state ^{13}C MAS NMR spectra were recorded on a AVANCE III 400 MHz WB solid-state NMR spectrometer (Bruker, USA). A HUAWEI Mate 30 rear camera was used to take photographs of the CN and NV_2-CN electrodes in Fig. 2b, Fig. 5e and Fig. S3. The international standards organization (ISO), aperture size, and shutter speed were set at 6400, F1.8, and 1/60, respectively.

Preparation of CN and NVx-CN

Pristine CN was synthesized according to the previously reported literature[1]. First, 5.0 g of urea was placed into a tube furnace (OTF-1200X-S, Hefei Kejing Materials Technology Co., Ltd, China) and calcined at 550 °C under N_2 for 4 h (heating rate 3 °C/min). After cooling down, the bulk agglomerates were ground into powder, and pristine CN was obtained.

NV_x-CN was synthesized as follows: 15 g of urea was dissolved into 30 mL of aqueous KOH solution, and then the resulting solution was evaporated to dryness in an oven at 80 °C overnight. The solid mixtures of urea and KOH were then calcined at 550 °C in a tube furnace under N_2 for 4 h using a heating rate of 3 °C/min. Products were denoted as NV_x-CN (where x represents the mass ratio of KOH used with 5 g urea, ‰).

Preparation of CN and NV_x-CN photoelectrode

50 mg of CN or NV_x-CN was ultrasonically dispersed in 100 mL of acetone for 3 h to form a relatively stable suspension of CN or NV_x-CN nanoparticles. The deposition ink, containing 10 mL of CN or NV_x-CN nanoparticle suspension and 3 μL of HI, was prepared for electrophoretic deposition onto FTO substrate. Briefly, two FTO electrodes with an active area of 1 × 1 cm^2 were placed in parallel 1.5 cm apart in the suspension, and a voltage of 25 V was applied for 10 min to deposit CN/NV_x-CN films.

ECL Measurements

The ECL intensity measurements were carried out on an ECL analyzer system (MPI-E, Xi'an Ruimai Analytical Instruments Co. Ltd., China). The voltage of the photomultiplier tube (PMT) for collecting the ECL signal was biased at 170 V during detection.

Relative ECL efficiency determination[2-4]: To compare ECL efficiency (Φ_{ECL}) with different luminophores, a facile $Ru(bpy)_3Cl_2/K_2S_2O_8$ aqueous system was used as a reference in this study. The ECL emission spectra were recorded by integrating CHI 400C with a Fluoromax-4FL spectrophotometer, where the slit width was 3 nm. Φ_{ECL} was defined as the ratio of the number of photons produced per electron transferred between the oxidized and reduced analyte species relative to that of $Ru(bpy)_3Cl_2/K_2S_2O_8$, using Eq (1)[5, 6].

$$\phi_{ECL} = \frac{\left(\frac{\int ECL\,dt}{\int Current\,dt}\right)_x}{\left(\frac{\int ECL\,dt}{\int Current dt}\right)_{st}} \times 100\% \qquad (1)$$

where “ECL” and “Current” represent integrated ECL photon numbers from the corrected ECL spectrum according to the count sensitivity of PMT at different light wavelengths and Faradaic electrochemical current values, respectively, “st” refers to the $Ru(bpy)_3Cl_2/K_2S_2O_8$ standard and “x” refers to the analyte. The potential was fixed at different potentials (-0.9 V to -1.5 V vs. Ag/AgCl) by chronoamperometry in 0.01 M phosphate buffer saline (pH 7.4) containing 15 mM $K_2S_2O_8$ and 0.1 M KCl.

Calculation of photon counts: In this work, the spectrofluorometer coupled potentiostat was used as a high-resolution ECL spectrum acquisition system[7]. As known, the recorded emission spectrum would be distorted by the response function of the PMT (sensitivity as a function of wavelength). In this sense, the variability in PMT’s sensitivity to ECL emission at different wavelengths should be calibrated. In addition, the distance from the CN/NV_x-CN photoelectrode to the PMT surface and the CN/NV_x-CN photoelectrode surface area was the same when collecting photons from the CN/NV_x-CN photoelectrode and $Ru(bpy)_3^{2+}$.

Calculation of electrons: Unlike the Faradaic current, the non-Faradaic current during an electrochemistry process does not contribute to the ECL generation and should be subtracted when determining the intrinsic Φ_{ECL}. In this work, the potential was fixed at different potentials (–0.9 V to –1.5 V vs. Ag/AgCl) by chronoamperometry instead of the CV curve when collecting the ECL emission spectrum. The charge consumed by Faraday processes, including $K_2S_2O_8$ and CN reduction in ECL, can be quantitatively evaluated by subtracting the charges consumed in electrolytes without $K_2S_2O_8$ from those with $K_2S_2O_8$. Lastly, at the beginning of the i-t curve for the ECL reaction, the current drops rapidly within a few seconds, corresponding to the charging current. It does not contribute to the ECL generation. Therefore, the electron should be calculated after the i-t curve reaches a plateau.

In-situ electrochemical impedance spectroscopy (EIS) measurements

The in-situ electrochemical impedance spectroscopy (EIS) measurements were recorded in a Reference 600 potentiostat/galvanostat/ ZRA (Gamry, USA). The potentials were measured against the Ag/AgCl (saturated KCl). EIS experiments were performed in a typical three-electrode system, consisting of CN or NV_x-CN photoelectrode, platinum wires, and Ag/AgCl in saturated KCl as the working electrode, counter electrode, and reference electrode, respectively. EIS tests were performed using 10 mV amplitude at different applied potentials versus Ag/AgCl in the frequency range of 0.1 to 100000 Hz. Electrolytes: 0.01 M phosphate buffer saline, 0.1 M KCl, and 15

mM $K_2S_2O_8$.

Photoelectrochemical measurements

All the electrochemical measurements were performed with a conventional three-electrode system, consisting of CN or NV_x-CN photoelectrode, platinum wires, Ag/AgCl in saturated KCl as the working electrode, counter electrode, and reference electrode, respectively. The open circus potential (OCP) was recorded in a Reference 600 potentiostat/galvanostat/ZRA (Gamry, USA). The potentials were measured against the Ag/AgCl (saturated KCl). The light source to simulate the sunlight was obtained from a 150 W Xe lamp and the average light intensity was 100 mW/cm^2.

Measurement time-resolved PL decay spectra

The Time-resolved PL decay spectra were recorded under different biased potentials ranging from –0.9 V to –1.5 V vs. Ag/AgCl to simulate the accumulation of electrons in NV_2-CN. A 360 nm pulsed excitation light was applied and the PL signal produced by CN or NV_2-CN was simultaneously detected. The experiments were performed in a typical three-electrode system, consisting of CN or NV_2-CN photoelectrode, platinum wires, and Ag/AgCl in saturated KCl as the working electrode, counter electrode, and reference electrode, respectively. Electrolytes contained 0.01 M phosphate buffer saline and 0.1 M KCl.

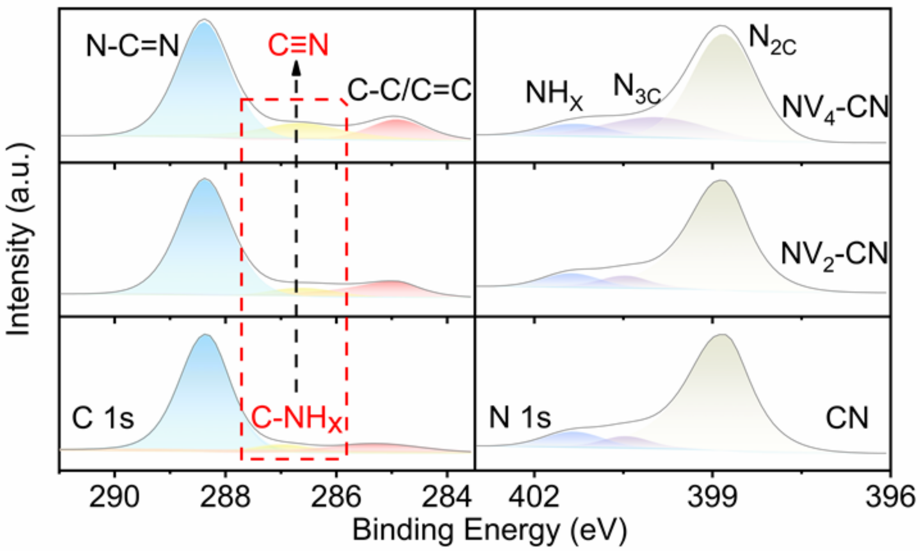

Fig. S1 High-resolution N1s and C1s XPS spectra of CN, NV_2-CN and NV_4-CN.

To further prove the trend that -C≡N increases with the increase of KOH content, we have analyzed the XPS C1s spectra of three different defect concentrations. As shown in Fig. S1, both two C 1s spectra of carbon nitride contain three peaks, located at 288.3 eV, 286.40 eV and 284.70 eV, corresponding to N-C=N, C-NH_x and amorphous carbon in the aromatic ring of carbon nitride respectively. -C≡N has similar binding energy as C-NH_x; the increase in binding energy at 286.40 eV with increasing defects verifies this trend. The high-resolution N 1s spectra can be deconvoluted into 4 peaks at 398.7 eV (attributed to sp^2 nitrogen bonding on nitrogen-containing aromatic rings, N_{2C}), 400.5 eV (N_{3C}), 401.4 eV (N-H_X) and 404.2 eV (charging effects in heterocycles or positive charge localization)[8].

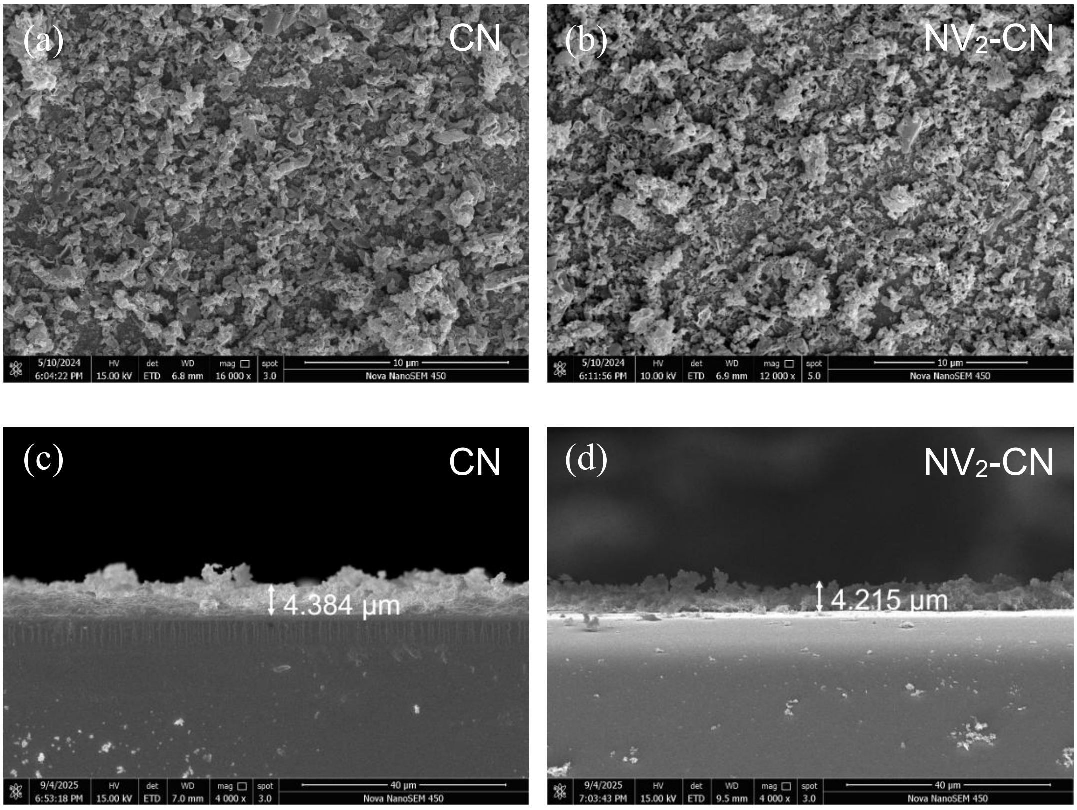

Fig. S2 Top and cross-sectional SEM images of CN (a and c) and NV_2-CN (b and d).

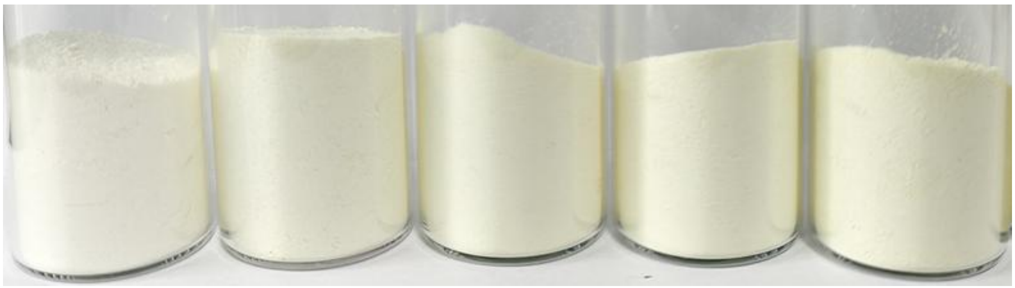

Fig. S3 Digital photographs of carbon nitrides prepared with different amounts of KOH (CN, $NV_{0.4}$-CN, NV_1-CN, NV_2-CN and NV_4-CN, from left to right).

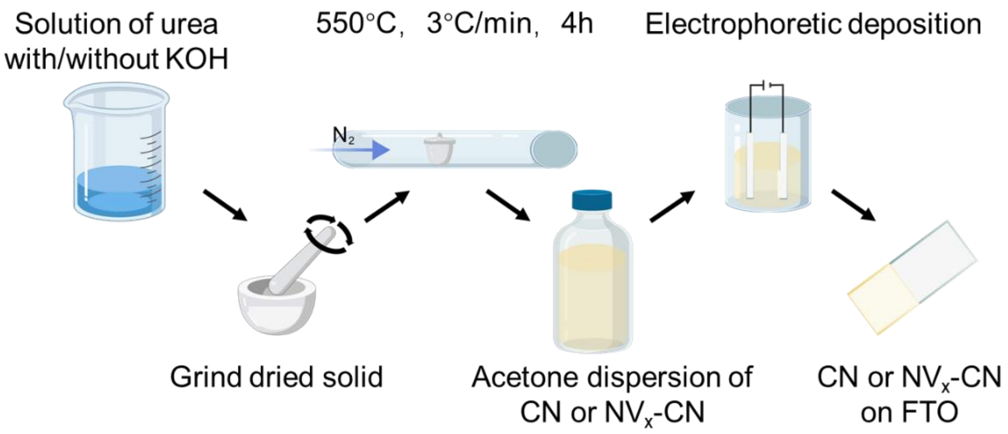

Fig. S4 Scheme of the general fabrication procedure for CN and NV_x-CN photoelectrode on the FTO glass.

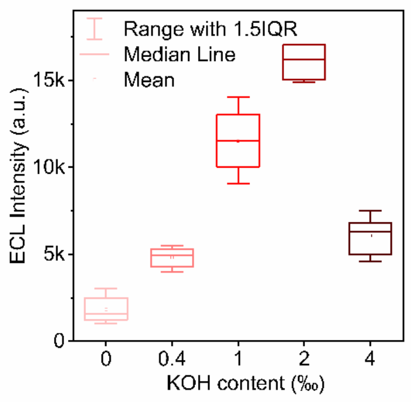

Fig. S5 ECL emission intensity of NV_x-CN photoelectrode with various KOH content. The boxplots display the interquartile range (boxes), the median (horizontal lines in the middle), the 25th and 75th percentile (horizontal lines in the bottom and top), and the mean (square), n = 5 per group.

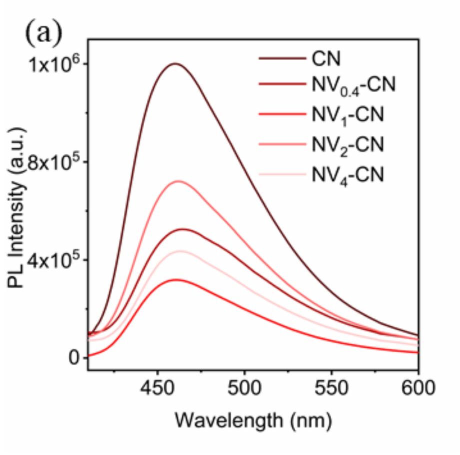

Fig. S6 PL spectra (a) and normalized PL spectra (b) of the CN, $NV_{0.4}$-CN, NV_1-CN, NV_2-CN and NV_4-CN.

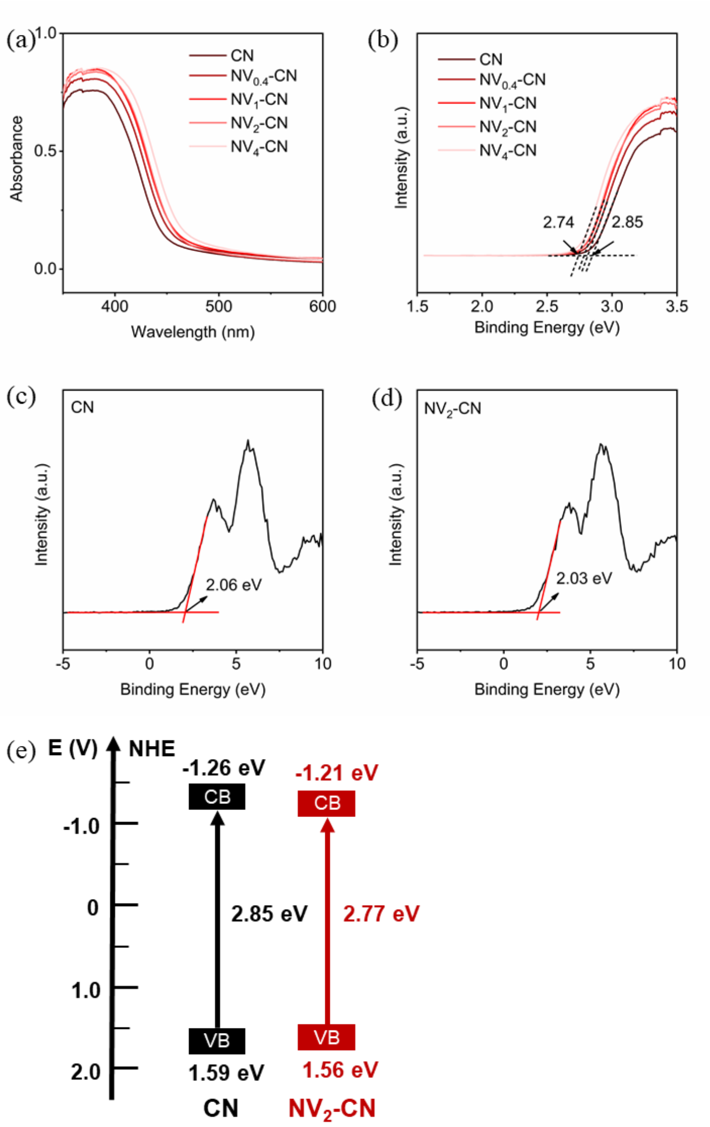

Fig. S7 UV-vis spectrum (a) and Kubelka-Munk plot (b) of CN, $NV_{0.4}$-CN, NV_1-CN, NV_2-CN and NV_4-CN. VB-XPS spectra of CN (c) and NV_2-CN (d). (e) Energy level diagram of CN and NV_2-CN.

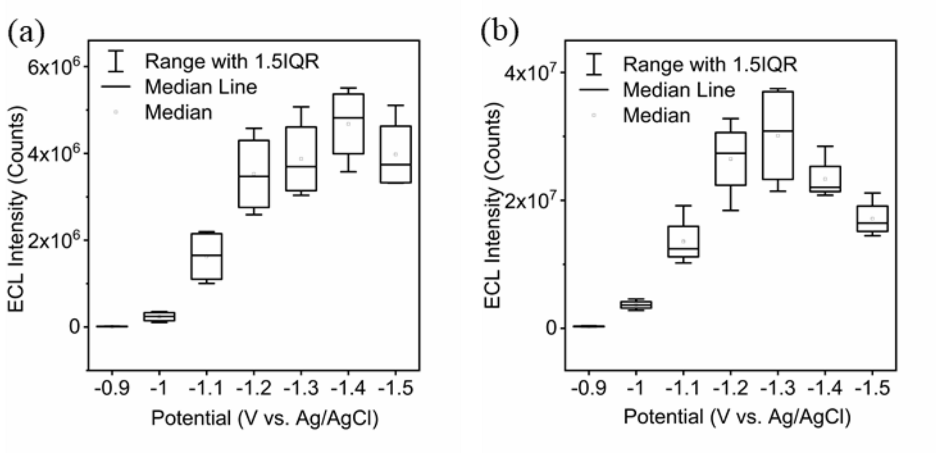

Fig. S8 ECL intensity of CN (a) and NV_2-CN (b) photoelectrodes at different potentials.

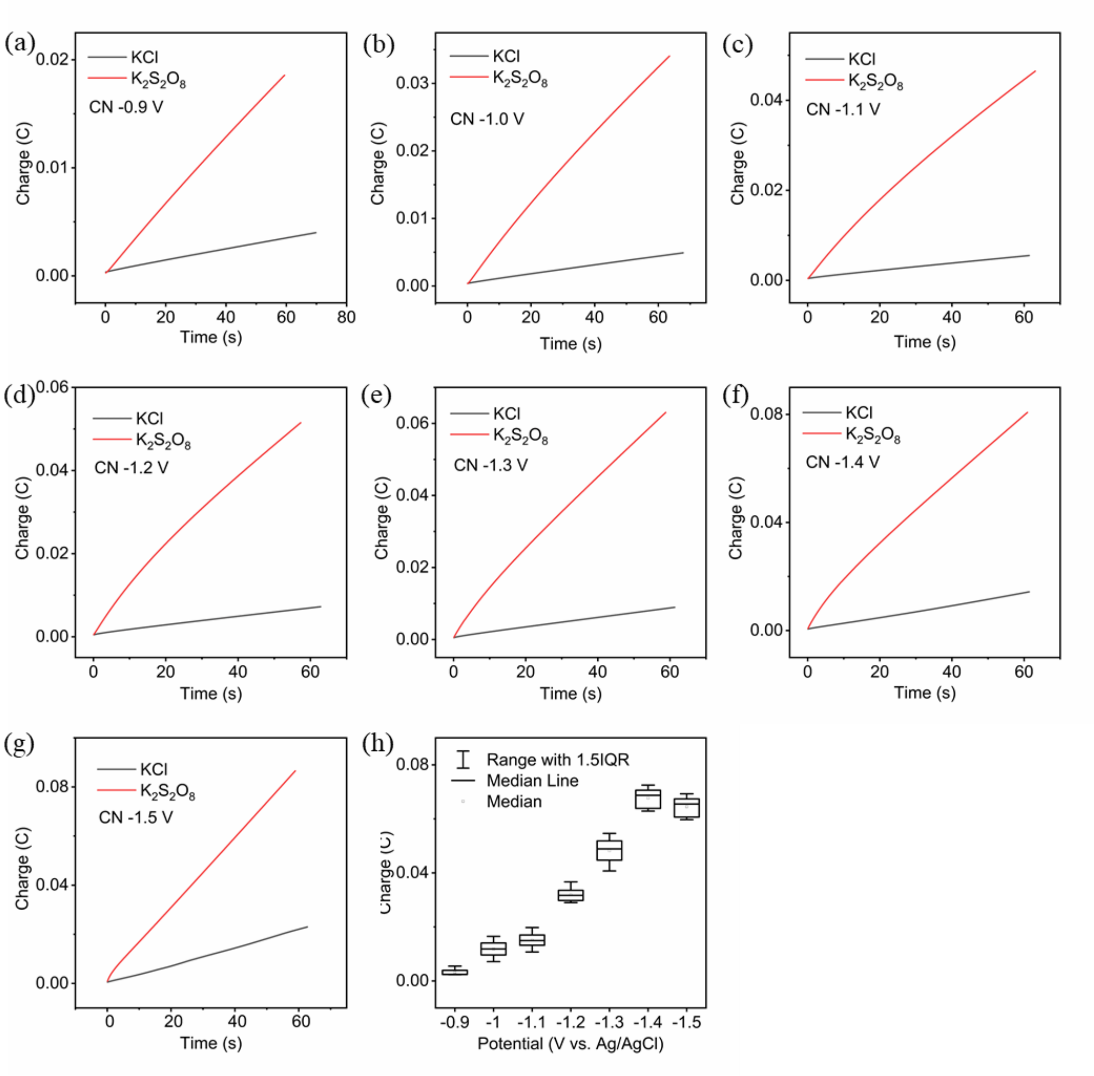

Fig. S9 Amperometric Q-t curve for ECL reaction of CN at –0.9 V (a), –1.0 V (b), –1.1 V (c), –1.2 V (d), –1.3 V (e), –1.4 V (f)and –1.5 V (g) in 0.01 M PBS, 0.1 M KCl with 15 mM $K_2S_2O_8$ (red line) and without $K_2S_2O_8$ (black line). (h) The number of charges consumed by Faraday reactions at different potentials (–0.9 V ~ –1.5 V).

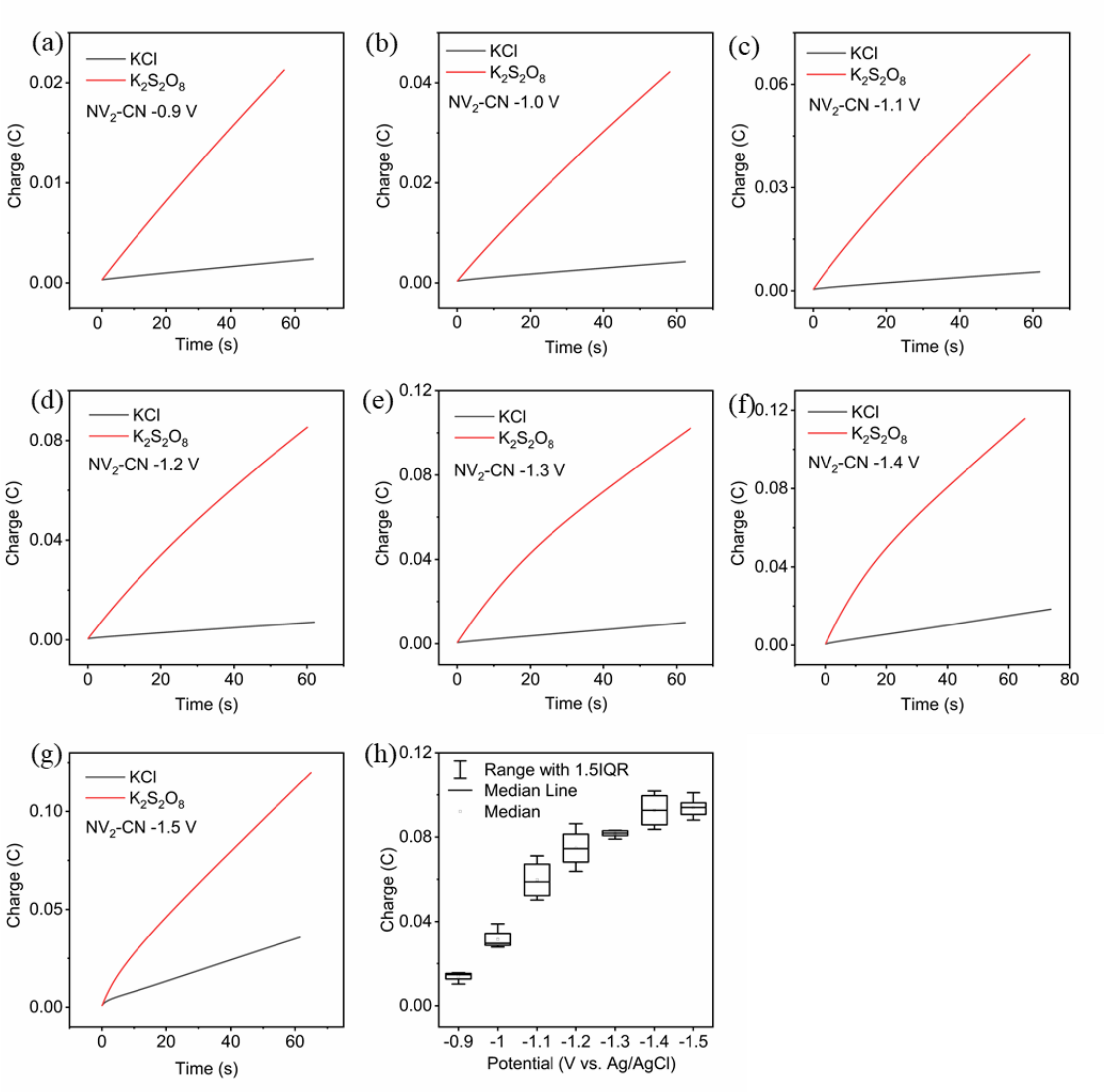

Fig. S10 Amperometric Q-t curve for ECL reaction of NV_2-CN at –0.9 V (a), –1.0 V (b), –1.1 V (c), –1.2 V (d), –1.3 V (e), –1.4 V (f) and –1.5 V (g) in 0.01 M PBS, 0.1 M KCl with 15 mM $K_2S_2O_8$ (red line) and without $K_2S_2O_8$ (black line). (h) The number of charges consumed by Faraday reactions at different potentials (–0.9 V ~ –1.5 V).

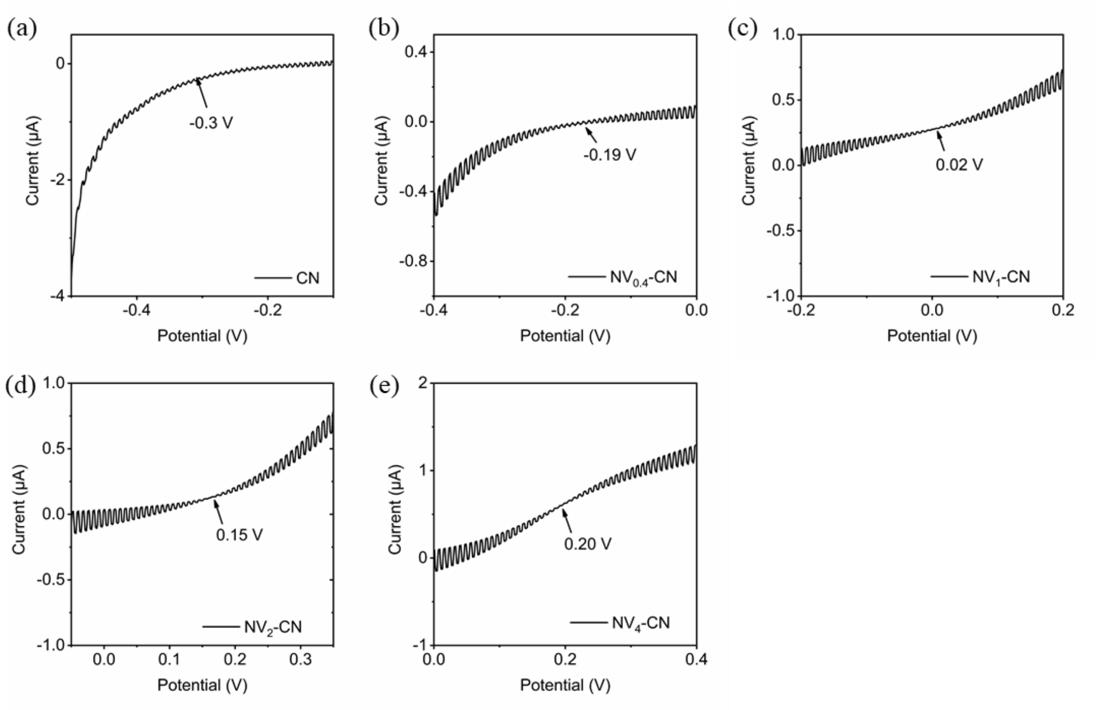

Fig. S11 Current–potential curves for the CN (a), $NV_{0.4}$-CN (b), NV_1-CN (c), NV_2-CN (d), and NV_4-CN (e) photoelectrodes under chopped irradiation. All the illuminated area of the photoelectrode is 1 cm^2. The photocurrent measurements were performed in 0.1 M KCl solution. The photocurrent was monitored while sweeping the potential in the negative direction, at a scan rate of 5 mV/s.

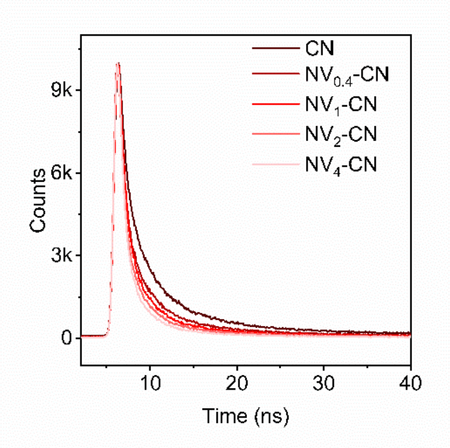

Fig. S12 Time-resolved PL decay spectra of CN, $NV_{0.4}$-CN, NV_1-CN, NV_2-CN and NV_4-CN under 360 nm excitation.

To quantitatively disclose the electron release kinetics, time-resolved PL decay spectra were measured. Three different processes lifetimes mediated by defects, including non-radiative process (τ_1), radiative process (τ_2), and energy transfer process (τ_3), can be obtained by fitting. Compared to CN, with the increase in defect concentration, the lifetimes of the three processes in NV_x-CN all decrease, indicating that a high defect density accelerated electron release. Thus, as the density of defect states increased, the ability of defects to capture electrons was enhanced; meanwhile an excessively high defect density would accelerate the release dynamics. Along this line, NV_2-CN that has moderate defect density exhibited the highest accumulate electrons.

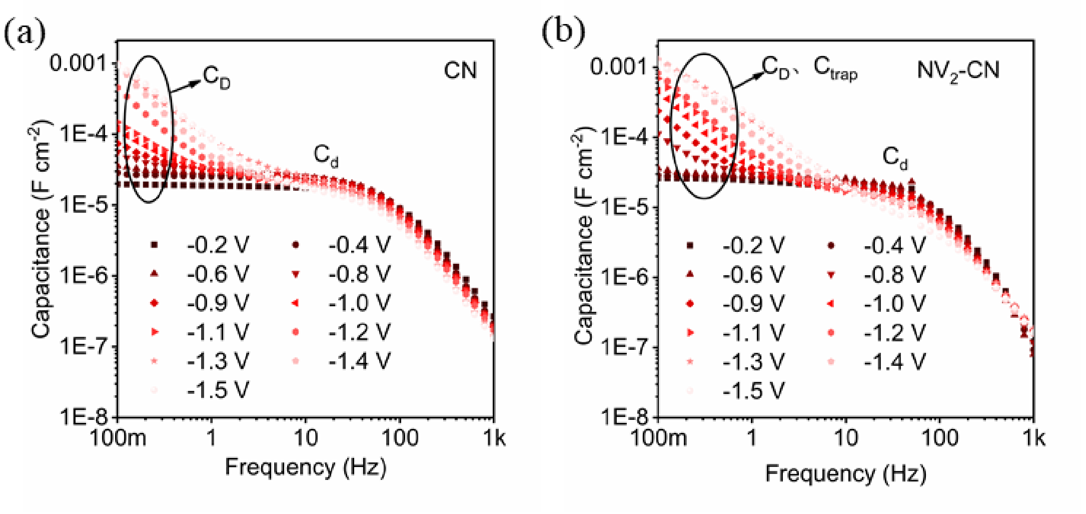

Fig. S13 Impedance diagrams (Frequency-C_{real}) measured at different potentials for CN (a) and NV_2-CN (b).

In the mid-to-high frequency range, the capacitance is composed of double-layer capacitance (C_d). At low frequencies, the capacitance is composed of diffusion layer capacitance (C_D)[9]. In the case of NV_x-CN with defects, a third chemical capacitance emerges at low frequencies as a result of electron accumulation, which is referred to here as defect state capacitance (C_{trap})[10]. The real part of the admittance capacitance (C_{real}) represents the actual capacitance value within an AC circuit, which indicates the ability of the capacitor to store energy. However, it is difficult to distinguish C_D and C_{trap}.

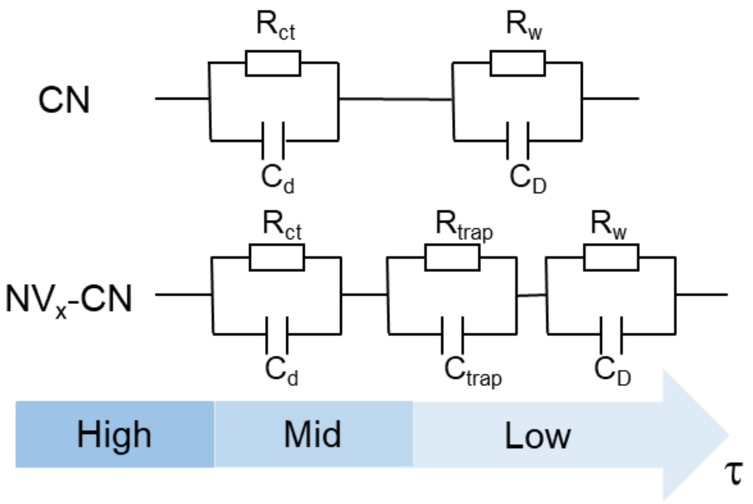

Fig. S14 Approximate equivalent circuit used for interpretation of CN and NV_x-CN photoelectrodes in DRT fitting.

DRT approximates the system impedance model by connecting infinitely many resistors (R) and capacitors (C) in parallel. Therefore, according to the literature and the existing DRT fitting results[2, 10], the approximate equivalent circuit with their main types of RC of CN and NV_x-CN are listed. Generally, the original CN contains two capacitances: double layer capacitance (C_d) controls the interfacial charge transfer resistance (R_{ct}) during the Faraday reaction at mid-frequencies, and the diffusion layer capacitance (C_D) controls the Warburg impedance (R_w) at low-frequencies. However, NV_x-CN had a third type of capacitance at low-frequencies, due to the accumulated electrons in the defects, which is referred to here as the trap capacitance (C_{trap}).

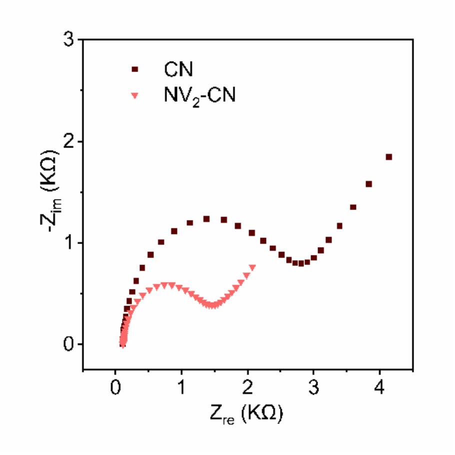

Fig. S15 Nyquist plots for CN and NV_2-CN in 0.1 M KCl containing 5 mM $K_3[Fe(CN)_6]/K_4[Fe(CN)_6]$.

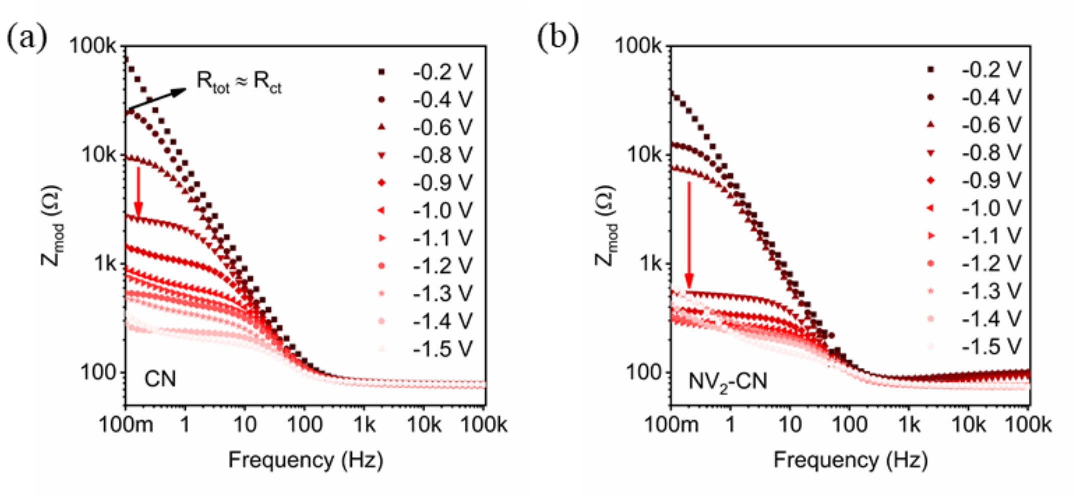

Fig. S16 Impedance diagrams (Frequency-Z_{mod}) measured at different potentials for CN (a) and NV_2-CN (b).

The impedance of the capacitor was close to 0 at high-frequency and approaches infinity at low-frequency. C_{sc} and C_d are in parallel with R_t and R_{ct}. When the capacitive impedance is high, the resistive impedance is the determining factor. In the low-frequency region of the graph, the phase angle approximates to 0º, indicating the combined resistances of R_s, R_t, and R_{ct}, collectively represented as R_{tot}. Typically, $R_{ct} > R_t > R_s$ and both R_t and R_s are intrinsic properties of the system that remain constant regardless of voltage changes. Therefore, it is considered that at this situation, R_{ct} approximates to R_{tot}. R_t represented the resistivity of electron transport in the emitter film. R_{ct} denoted the charge reaction resistance at the ECL emitter/$S_2O_8^{2-}$ interface, which was caused by the Faradaic reaction. R_s represented the solution resistance.

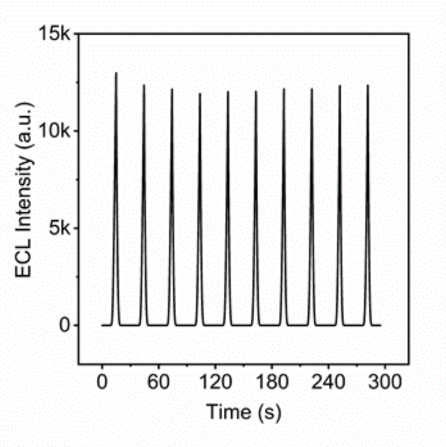

Fig. S17 ECL intensity of NV_2-CN based biosensor used to detect NO_2^- under consecutive cyclic potential scanning. “a.u.” refers to arbitrary units.

Table S1 Elemental analysis using the XPS data.

	C/N	O/C
CN	0.900	0.046
NV_2-CN	0.912	0.053

Table S2 Elemental analysis (EA) results of CN, NV_2-CN and NV_4-CN.

Atomic radio	C/N	O/C
CN	0.562	0.028
NV_2-CN	0.569	0.009
NV_4-CN	0.578	N.D.

Table S3 Proportion of N_{2C}、N_{3C}、N-H_X and charging effect in CN and NV_2-CN according to XPS N1s analysis. .

Sample	N_{2C}	N_{3C}	N-H_x	Charging Effect
CN	81.50	9.66	6.18	2.67
NV_2-CN	81.37	9.56	7.21	2.75

Table S4 Fitted τ_1, τ_2, and τ_3 using the Time-resolved PL decay spectra of CN, $NV_{0.4}$-CN, NV_1-CN, NV_2-CN and NV_4-CN under 360 nm excitation.

Samples	τ_1 (ns)	τ_2 (ns)	τ_3 (ns)
CN	0.954	4.015	18.272
$NV_{0.4}$-CN	0.764	3.367	17.104
NV_1-CN	0.747	3.138	16.672
NV_2-CN	0.706	2.927	15.756
NV_4-CN	0.641	2.641	15.085

Table S5 Fitting impedance data for CN and NV_2-CN photoelectrode in 0.01 M phosphate buffer saline containing 0.1 M KCl and 15 mM $K_2S_2O_8$ at different potential.

Potential (V)	NV_2-CN				CN		
	R_{ct} (ohm)	C_d (μF)	C_{trap} (μF)	C_D (μF)	R_{ct} (ohm)	C_d (μF)	C_D (μF)
–0.8	412.7	20.40	32.86	36.17	2353.18	22.72	24.06
–0.9	245.85	18.17	55.38	67.80	1046.42	21.34	44.14
–1	180.8	15.87	117.08	219.85	534.52	18.75	77.51
–1.1	153.25	14.88	152.21	350.81	412.59	16.90	106.5
–1.2	141.82	14.73	155.68	456.37	348.38	18.59	321.21
–1.3	120.73	14.61	160.92	904.12	266.1	20.54	669.46
–1.4	127.83	15.32	134.61	784.98	157.43	16.11	710.89
–1.5	83.75	11.46	124.04	906.39	125.8	14.21	778.89

According to the following formula[12-15],

$$Z_{DRT} = R_\infty + \int_{-\infty}^{\infty} \frac{\gamma(ln\tau)}{1+i2\pi f\tau} dln\tau \approx R_\infty + \int_{ln\tau_0}^{ln\tau_M} \frac{\sum_{m=1}^{M} x_m \varphi_m(ln\tau)}{1+i2\pi f\tau} dln\tau \quad (2)$$

$$C(\omega) = C_{eff} \int_{-\infty}^{\infty} \frac{\Gamma(log\,(\tau))}{1+i\omega\tau} d(log\,(\tau)) \quad (3)$$

with ln(τ) as the abscissa, integration can be performed to obtain the impedance of each peak. Similarly, with log(τ) as the abscissa, each normalized peak integration is multiplied by the effective capacitance that extracted from the impedance diagrams (Frequency-Creal), to determine its respective contributions to the total capacitance.[16].

The attribution of the second and the third DRT peaks in the NV_2-CN system is discussed as follows. Although both trends are consistent with the variation characteristics described by the formula that the defect capacitance first increases and then decreases with the voltage. Nevertheless, the fitting values in Supplementary Table 4 indicate an increase in the capacitance of the third DRT peak at -1.5V. This result is caused by accelerated interfacial reaction driven by the high voltage. Moreover, the values of the third DRT peak in the NV_2-CN system are similar to that of the second DRT peak in the CN system, indicating these peaks can all be ascribed to C_D. Besides, the second DRT peak in the NV_2-CN system is closer to the C_{trap} reported in the literature in terms of relaxation time and order of magnitude[17-19]. Therefore, for the NV_2-CN system, the second DRT peak is attributed to C_{trap}, while the third DRT peak is attributed to C_D.

Table S6 Fitted τ_1, τ_2, τ_3, and τ_{ave} using the Time-resolved PL decay spectra of CN and NV_2-CN under 360 nm excitation at different potential.

Potential (V)	CN				NV_2-CN			
	τ_1 (ns)	τ_2 (ns)	τ_3 (ns)	τ_{ave} (ns)	τ_1 (ns)	τ_2 (ns)	τ_3 (ns)	τ_{ave} (ns)
–0.8	7.21	31.66	1.74	4.38	4.86	29.52	1.15	2.58
–0.9	6.27	29.76	1.71	4.21	4.69	29.60	1.11	2.38
–1.0	7.02	34.66	1.86	4.33	4.67	31.50	1.00	2.36
–1.1	6.75	31.95	1.82	4.29	4.76	30.50	1.13	2.36
–1.2	6.31	31.29	1.68	4.27	4.26	28.05	0.97	2.22
–1.3	6.71	30.25	1.70	4.31	4.14	27.59	0.94	2.19
–1.4	6.28	32.59	1.91	4.36	4.67	32.56	1.04	2.35
–1.5	6.54	31.25	1.70	4.19	4.58	30.58	1.03	2.33

Discussion about higher electron mobility in defective CN

Notably, the fact of higher electron mobility in defective CN seems counterintuitive. It should be clarified that the defect concentration of NV_2-CN prepared in this study is at an extremely low level, thus the situation where defects hinder electron transfer does not apply to this system. Indeed, numerous previous studies have documented the enhancing effect of defects on electron transfer under specific conditions [20, 21]. As confirmed by the characteristic signal of ESR (**Fig. 1e**), the introduction of defects may significantly increase the charger carrier concentration, which would further lead to the decrease of charge transfer resistance at the electrode interface. This deduction is well consistent with the trend of arc radius changes in the Nyquist plots (**Fig. 3e**).

Supplementary references

[1] Yu, H.; Shi, R.; Zhao, Y.; Bian, T.; Zhao, Y.; Zhou, C.; Waterhouse, G. I. N.; Wu, L. Z.; Tung, C. H.; Zhang, T. Alkali-Assisted Synthesis of Nitrogen Deficient Graphitic Carbon Nitride with Tunable Band Structures for Efficient Visible-Light-Driven Hydrogen Evolution. *Adv. Mater.* **2017**, *29*, 1605148.

[2] Fang, Y.; Yang, H.; Hou, Y.; Li, W.; Shen, Y.; Liu, S.; Zhang, Y. Timescale correlation of shallow trap states increases electrochemiluminescence efficiency in carbon nitrides. *Nat. Commun.* **2024**, *15*, 3597.

[3] Hou, Y.; Fang, Y.; Zhou, Z.; Hong, Q.; Li, W.; Yang, H.; Wu, K.; Xu, Y.; Cao, X.; Han, D.; Liu, S.; Shen, Y.; Zhang, Y. Growth of Robust Carbon Nitride Films by Double Crystallization with Exceptionally Boosted Electrochemiluminescence for Visual DNA Detection. *Adv. Opt. Mater.* **2023**, *11*, 2202737.

[4] Zhao, T.; Zhou, Q.; Lv, Y.; Han, D.; Wu, K.; Zhao, L.; Shen, Y.; Liu, S.; Zhang, Y. Ultrafast Condensation of Carbon Nitride on Electrodes with Exceptional Boosted Photocurrent and Electrochemiluminescence. *Angew. Chem. Int. Ed.* **2020**, *59*, 1139-1143.

[5] Adsetts, J. R.; Chu, K.; Hesari, M.; Ma, J.; Ding, Z. Absolute Electrochemiluminescence Efficiency Quantification Strategy Exemplified with $Ru(bpy)_3^{2+}$ in the Annihilation Pathway. *Anal. Chem.* **2021**, *93*, 11626-11633.

[6] Zhang, R.; Adsetts, J. R.; Nie, Y.; Sun, X.; Ding, Z. Electrochemiluminescence of nitrogen- and sulfur-doped graphene quantum dots. *Carbon* **2018**, *129*, 45-53.

[7] Lv, Y.; Zhou, Z.; Shen, Y.; Zhou, Q.; Ji, J.; Liu, S.; Zhang, Y. Coupled Fluorometer-Potentiostat System and Metal-Free Monochromatic Luminophores for High-Resolution Wavelength-Resolved Electrochemiluminescent Multiplex Bioassay. *ACS Sens.* **2018**, *3*, 1362-1367.

[8] Wang, Y.; Du, P.; Pan, H.; Fu, L.; Zhang, Y.; Chen, J.; Du, Y.; Tang, N.; Liu, G. Increasing Solar Absorption of Atomically Thin 2D Carbon Nitride Sheets for Enhanced Visible-Light Photocatalysis. *Adv. Mater.* **2019**, *31*, 1807540.

[9] Xie, C.; Chen, W.; Du, S.; Yan, D.; Zhang, Y.; Chen, J.; Liu, B.; Wang, S. In-situ phase transition of WO_3 boosting electron and hydrogen transfer for enhancing hydrogen evolution on Pt. *Nano Energy* **2020**, *71*, 104653.

[10] Zarazua, I.; Han, G.; Boix, P. P.; Mhaisalkar, S.; Fabregat-Santiago, F.; Mora-Seró, I.; Bisquert, J.; Garcia-Belmonte, G. Surface Recombination and Collection Efficiency in Perovskite Solar Cells from Impedance Analysis. *J. Phys. Chem. Lett.* **2016**, *7*, 5105-5113.

[11] Wang, Q.; Moser, J. E.; Gratzel, M. Electrochemical impedance spectroscopic analysis of dye-sensitized solar cells. *J. Phys. Chem. B* **2005**, *109*, 14945-14953.

[12] Wan, T. H.; Saccoccio, M.; Chen, C.; Ciucci, F. Influence of the Discretization Methods on the Distribution of Relaxation Times Deconvolution: Implementing Radial Basis Functions with DRTtools. *Electrochim. Acta* **2015**, *184*, 483-499.

[13] Ciucci, F.; Chen, C. Analysis of Electrochemical Impedance Spectroscopy Data Using the Distribution of Relaxation Times: A Bayesian and Hierarchical Bayesian Approach. *Electrochim. Acta* **2015**, *167*, 439-454.

[14] Effat, M. B.; Ciucci, F. Bayesian and Hierarchical Bayesian Based Regularization for Deconvolving the Distribution of Relaxation Times from Electrochemical Impedance Spectroscopy Data. *Electrochim. Acta* **2017**, *247*, 1117-1129.

[15] Liu, J.; Wan, T. H.; Ciucci, F. A Bayesian view on the Hilbert transform and the Kramers-Kronig transform of electrochemical impedance data: Probabilistic estimates and quality scores. *Electrochim. Acta* **2020**, *357*, 136864.

[16] Oz, A.; Hershkovitz, S.; Belman, N.; Tal-Gutelmacher, E.; Tsur, Y. Analysis of impedance spectroscopy of aqueous supercapacitors by evolutionary programming: Finding DFRT from complex capacitance. *Solid State Ionics* **2016**, *288*, 311-314.

[17] Klahr, B.; Gimenez, S.; Fabregat-Santiago, F.; Bisquert, J.; Hamann, T. W. Electrochemical and photoelectrochemical investigation of water oxidation with hematite electrodes. *Energy Environ. Sci.* **2012**, *5*, 7626-7636.

[18] Upul Wijayantha, K. G.; Saremi-Yarahmadi, S.; Peter, L. M. Kinetics of oxygen evolution at alpha-Fe_2O_3 photoanodes: a study by photoelectrochemical impedance spectroscopy. *Phys. Chem. Chem. Phys.* **2011**, *13*, 5264-5270.

[19] Klahr, B.; Gimenez, S.; Fabregat-Santiago, F.; Hamann, T.; Bisquert, J. Water oxidation at hematite photoelectrodes: the role of surface states. *J. Am. Chem. Soc.* **2012**, *134*, 4294-4302.

[20] Klahr, B.; Gimenez, S.; Fabregat-Santiago, F.; Hamann, T.; Bisquert, J. "Water oxidation at hematite photoelectrodes: the role of surface states" *J. Am. Chem. Soc.* **2012**, 134, 4294-4302.

[21] Klahr, B.; Gimenez, S.; Fabregat-Santiago, F.; Bisquert, J.; Hamann, T. W. "Electrochemical and photoelectrochemical investigation of water oxidation with hematite electrodes" *Energy Environ. Sci.* **2012**, 5, 7626-7636.
